# Optimized Decellularization Protocol for Large Peripheral Nerve Segments: Towards Personalized Nerve Bioengineering

**DOI:** 10.3390/bioengineering9090412

**Published:** 2022-08-24

**Authors:** Alois Hopf, Lina Al-Bayati, Dirk J. Schaefer, Daniel F. Kalbermatten, Raphael Guzman, Srinivas Madduri

**Affiliations:** 1 Department of Biomedical Engineering, University of Basel, Gewerbestrasse 14, 4123 Allschwil, Switzerland; 2 Department of Biomedicine, University Hospital Basel, Hebelstrasse 20, 4031 Basel, Switzerland; 3 Department of Plastic, Reconstructive, Aesthetic and Hand Surgery, University Hospital Basel, University of Basel, Spitalstrasse 21, 4031 Basel, Switzerland; 4 Plastic, Reconstructive and Aesthetic Surgery, Department of Surgery, Geneva University Hospitals, University of Geneva, 1211 Geneva, Switzerland; 5 Bioengineering & Neuroregeneration, Department of Surgery, Geneva University Hospitals, University of Geneva, Rue Michel-Servet 1, 1211 Geneva, Switzerland; 6 Department of Neurosurgery, University Hospital Basel, Spitalstrasse 21, 4031 Basel, Switzerland

**Keywords:** decellularization, acellular allograft, large-gap repair, neurotmesis, peripheral nerve injuries, porcine sciatic nerve

## Abstract

Nerve injuries remain clinically challenging, and allografts showed great promise. Decellularized nerve allografts possess excellent biocompatibility and biological activity. However, the vast majority of decellularization protocols were established for small-size rodent nerves and are not suitable for clinical application. We aimed at developing a new method of decellularizing large-diameter nerves suitable for human transplantation. Repeated rounds of optimization to remove immunogenic material and preserve the extracellular structure were applied to the porcine sciatic nerve. Following optimization, extensive in vitro analysis of the acellular grafts via immunocytochemistry, immunohistology, proteomics and cell transplantation studies were performed. Large segments (up to 8 cm) of the porcine sciatic nerve were efficiently decellularized and histology, microscopy and proteomics analysis showed sufficient preservation of the extracellular matrix, with simultaneous consistent removal of immunogenic material such as myelin, DNA and axons, and axonal growth inhibitory molecules. Cell studies also demonstrated the suitability of these acellular grafts for 3D cell culture studies and translation to future large animal studies and clinical trials. By using non-human donors for peripheral nerve transplantation, significant drawbacks associated with the gold standard can be eliminated while simultaneously preserving the beneficial features of the extracellular matrix.

## 1. Introduction

Peripheral nerve injuries are of clinical importance, with an estimated global incidence of one million cases each year [1]. Lesions in the peripheral nerve result in a decreased or complete loss of sensory and motor functions. Although it was recognized more than a century ago that peripheral nerves possess a regenerative capacity, clinical outcomes of therapies are often unsatisfactory, especially in severe injuries [2]. Direct suturing is promising for small injuries, but only because satisfactory recovery requires tension-free nerve reconstruction [3,4]. Following injuries with extensive damage, including a substantial loss of nerve tissue, autologous nerve transplantation is the current gold standard [5]. However, only around 50% of patients who receive autografts regain normal function [6] due to its limitations, such as a limited supply, donor-site morbidity, and size-modality mismatch. Thus, there is a need to find new innovative therapies [7,8]. Over decades of research, a wide variety of available transplants have been developed and range from synthetic biocompatible conduits to autologous non-nervous tissue grafts, such as veins or muscles, to processed allografts and autografts [3,9]. One promising alternative to the current gold standard is processed allografts.

Allografts have the advantage of mitigating donor-site morbidity and are unlimited in supply and size. Furthermore, based on their extracellular matrix (ECM) composition, they are bioactive and biodegradable. By processing, allografts can be decellularized and therefore the immunogenic material, such as cells, cellular components and other regeneration inhibitors, can be removed from the tissue, with only the ECM scaffold remaining, preferably in its native structure and composition. By this method, the bioactive and mechanical properties of the scaffold remain, which was shown to induce cell adhesion, migration, proliferation, differentiation and angiogenesis [10]. However, the optimal balance between the structural integrity of the basal lamina and the ECM and the removal of cellular components and inhibitors remains to be defined.

Multiple methods have been developed for preparing decellularized nerve grafts such as mechanical disruption, e.g., freeze-thawing, mainly at the beginning of the process in order to disrupt cell membranes and lyse the cells. More recently, decellularization of nerve grafts was accomplished using additional ultrasonication steps for improved debris removal, chemical detergents (ionic, non-ionic, zwitterionic), acid and alkaline treatments, hypo- and hypertonic solutions and chelating agents to lyse cells and solubilize cellular proteins, enzymatic degradation for the specific removal of inhibiting factors, and additional removal of cellular components and irradiation to disrupt proteins [8,11,12,13,14]. Various common decellularization methods and agents were reviewed by Gilpin and Yang in 2017 [15].

The ultimate goal of all these techniques is first to reduce graft immunogenicity by removing host cells and cellular components, myelin and axons. Second, to preserve the extracellular matrix and basal lamina, which support nerve regeneration [16]. Third, to remove inhibitory factors such as myelin and proteoglycan present in the native nerve [17,18]. However, most protocols were established for small-scale rat and mouse sciatic nerves and are therefore not suitable for human-size nerve segments. Decellularization is generally performed on a rolling shaker to expose the tissue to the decellularization agent with uniform and consistent concentrations. Thus, the main mode of penetrating the tissue is diffusion. As observed in this study, decellularization protocols established for rodent sciatic nerves were not suitable for the decellularization of large-diameter pig nerves and require several adjustments in detergents, concentrations and duration of exposure. 

**Figure 1:**
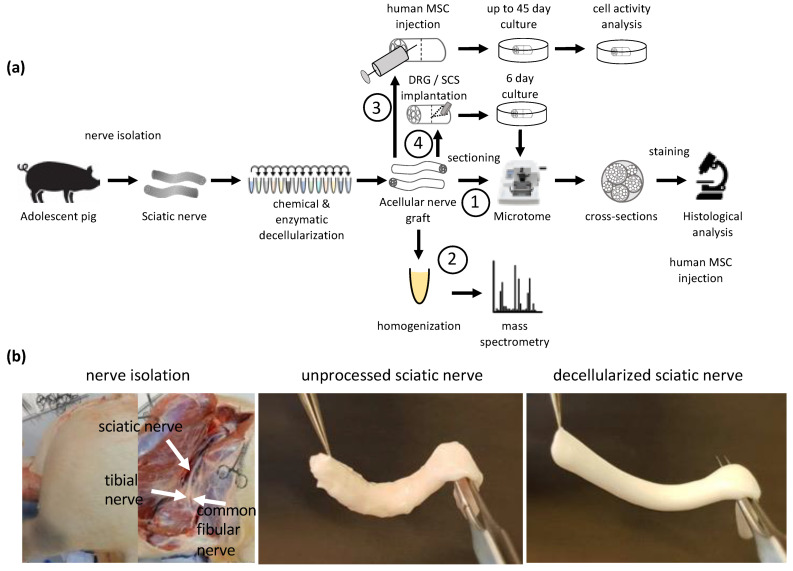
Experimental overview: (**a**) Illustration of the experimental setup. Sciatic and common fibular nerves of adolescent pigs were isolated. Following isolation, the nerves were treated with a series of various chemical and enzymatic detergents for decellularization. The resulting acellular nerve grafts were analyzed and tested in different ways. (1) Cross-sections via microtome were made from acellular graft and used for immunohistochemistry. (2) Proteomic analysis of the acellular graft to detect the number of removed peptides and known inhibitors of axonal regeneration due to the decellularization process. (3) Acellular grafts were used as 3D scaffolds for cell culture. Cells in the 3D scaffold were cultured for up to 45 days and cellular behavior was analyzed by measuring cellular metabolism via resazurin. (4) Dorsal root ganglion (DRG) and Spinal cord segments (SCS) were implanted in acellular grafts and cultured for 6 days prior to fixation, sectioning and histological analysis; (**b**) Representative images of nerve isolation from adolescent pigs. Unprocessed sciatic nerve with excessive fat and connective tissue and a reddish color, Decellularized sciatic nerve with removed excessive tissue and white appearance.

The goal of this study was to develop and investigate a new method of decellularizing large-diameter nerves, which are suitable for human transplantation. By combining a mild chemical disruption of cellular components with enzymatic degradation, a large acellular nerve scaffold was produced that does not contain detectable amounts of myelin or axons and has a preserved ECM structure. Injecting primary, human adipose-derived MSC (hASC) and tracking their activity allowed us to conclude upon the biocompatibility of these scaffolds (Figure 1).

## 2. Materials and Methods

### 2.1. Sciatic and Common Fibular Nerve Isolation

Nerve tissue was carefully isolated from domestic pigs (sus scrofa domesticus; Schweizer Edelschwein), which were euthanized 2–4 h prior to isolation. Special attention was given not to damage (pull and/or quench) the nerve and therefore not to disrupt the structure. The isolated nerve was sectioned in length from 0.5 cm to 8 cm for decellularization and subsequent analysis and testing. Tissue was collected in Phosphate-Buffered saline without Ca^2+^ and Mg^2+^ (PBS^−/−^) (Sigma-Aldrich, Buchs, Switzerland, Cat. No. D8537) on ice and delivered to the laboratory for experimentation. Unprocessed control tissue was placed immediately after isolation in 4% paraformaldehyde (PFA) (Carl Roth, Cat. No. 0335.2) in PBS^−/−^ and placed on ice for delivery to the laboratory where it was placed at 4 °C for the remaining 24 h of tissue fixation. 

No animals were raised or euthanized for the sole purpose of this study. For this study, biological waste was reused. Nerve tissue was isolated from 11 pigs post-mortem at the Center for Surgical Research & Central Biological Laboratory, University of Zurich. Animals were euthanized either by 100 mg/kg potassium chloride or by bleeding out under full anaesthesia. The animal housing and experimental protocols were approved by the Cantonal Veterinary Office, Zurich, Switzerland, under License ZH 115/2018, 084/2017, ZH 047/2020, ZH 052/2020, ZH 132/2017, ZH 213/2019 and ZH 219/2016.

### 2.2. Decellularization

Decellularization (DC) protocols were optimized based on previously published protocols for the decellularization of rodent nerves [7,16,19,20]. Protocol 1 was initially optimized for the decellularization of Sprague-Dawley rat sciatic nerves and subsequently applied to the nerves isolated from domestic pigs of 30 to 110 kg. Quantification experiments were performed using nerve segments of 4 cm and 8 cm, respectively. For cell transplantation studies, decellularization was performed on about 8 cm-long common fibular nerves, which were segmented in a size range of 4 mm after decellularization. All decellularization steps were performed at room temperature (RT) on an rs-tr05 roller mixer (Carl Roth, Karlsruhe, Germany) at 20 RPM unless otherwise stated. Segments were submerged in an excessive volume of detergents unless otherwise stated. After decellularization, each segment was washed 3 times to remove residual detergents in PBS^−/−^, each for 5 min. For the decellularization process, osmotic pressure, chemical disruption, solubilization and enzymatic degradation were applied for the removal of cells, cell debris, DNA, lipids, other immunogenic material and growth inhibitory molecules as described in more detail in the Section 4. 

In brief, the initial protocol (protocol 1) was as follows: nerve segments were placed in ddH_2_O for 7 h, followed by 1 M NaCl (Sigma-Aldrich, Cat. No. S7653) for 15 h. After rinsing nerve segments with ddH_2_O, segments were placed in 2.5 mM of non-ionic Span20 (Sigma-Aldrich, Cat. No. S6635) in PBS^−/−^ for 24 h. Span20 removal was performed by washing the segments in PBS^−/−^ for 15 min. Segments were then placed in ddH_2_O for 7 h, followed by 1 M NaCl for 15 h, and then rinsed in ddH_2_0. Next, segments were placed in 100 mM of zwitterionic CHAPS (Sigma-Aldrich, Cat. No. C3023) in PBS^−/−^ for 24 h. 

Protocol 2 followed the same procedure as protocol 1. However, at the end, nerve segments were placed in 1 mL/cm of segment length of 0.2 U/mL chondroitinase ABC (Merck, Tomeguro, Tokyo, Cat. No. C2905) in chondroitinase buffer (50 mM TRIS, 60 mM sodium acetate pH 8, 0.02% bovine serum albumin [BSA] (Sigma-Aldrich, Cat. No. A3294)) at 37 °C for 24 h. Segments were then rinsed in PBS^−/−^ and placed in 0.0 5 U/mL elastase (Merck, Cat. No. E7885) in PBS ^−/−^ for a further 24 h. 

Protocol 3 was based on protocol 2, but incubation times for non-ionic Span20 and zwitterionic CHAPS were doubled from 24 h to 48 h. All other incubation times remained the same and detergents and their order of applications were unchanged.

Protocol 4 was based on protocol 3. However, after initial decellularization steps in ddH_2_0 for 7 h and 1 M NaCl for 15 h, segments were placed in 4% sodium deoxycholate (Sigma-Aldrich, Cat. No. D6750) in ddH_2_O for 24 h.

Protocol 5, which was further used in the detailed analysis, was based on protocol 4, with the difference that sodium deoxycholate concentrations were reduced from 4% to 0.001%. 

### 2.3. Neurospecimen Preparation

Decellularization protocol optimization was performed on 1 cm-long segments of sciatic nerves. Quantification of remaining myelin, DNA, axonal debris and laminin was performed on 4 cm and 8 cm-long segments of decellularized sciatic nerve tissue. hASC were transplanted in 4 mm-long segments of decellularized common fibular nerves. Dorsal root ganglion (DRG) and spinal cord segment (SCS) were transplanted into 8 mm-long segments of decellularized sciatic nerves. Following decellularization, acellular grafts were either fixed immediately or stored at 4 °C in PBS^−/−^ overnight for cell transplantation. For proteomics studies, unprocessed tissue was stored at 4 °C in PBS^−/−^ for 11 days until the decellularization procedure of the experimental condition was completed. For the structural analysis, samples were fixed in 4% PFA at 4 °C for 24 h. The fixed segments were sequentially dehydrated in increasing EtOH concentrations and xylene and embedded in paraffin using a TPC 15 DUO (MEDITE GmbH, Dietikon, Switzerland). The 5 μm-thin cross- and longitudinal sections were made by a rotary microtome (Microm HM 340E; Thermo Fisher Scientific, Waltham, MA, USA).

### 2.4. Immunohistochemistry

Antigen retrieval by proteinase K (Sigma-Aldrich, Cat. No. 03115836001) for 10 min at 37 °C was performed prior to immunohistochemistry for polyclonal laminin staining. Sections were blocked for 1 h at RT in a blocking buffer consisting of 1% BSA and 0.1% TritonX-100 in PBS^−/−^. Primary antibodies, namely laminin (Merck, Cat. No. L9393), myelin basic protein (MBP) (Bio-Rad, Hercules, CA, USA: Cat. No. MCA409S) and β-tubulin III (Abcam, Chuo-ku, Tokyo, Cat. No. ab18207), were incubated overnight at 4 °C in the blocking buffer. Sections were then washed in PBS^−/−^ 3 times for 10 min before the addition of secondary antibodies (goat anti-rabbit IgG-a488; Thermo Fisher Scientific, Cat. No. A-11008; goat anti-rat IgG-a647; Thermo Fisher Scientific, Cat. No. A-21247) and 4′,6-diamidino-2-phenylindole (DAPI) (Sigma-Aldrich, Cat. No. 32670) in blocking buffer for 1 h at RT in the dark. After washing 3 times for 10 min with PBS^−/−^, the slides were covered with mounting media and coverslips. For a general evaluation of successful decellularization, longitudinal and cross-sections were further dyed using standard Gill’s hematoxylin and eosin protocol, staining acidic structures purple and basic structures pink. To verify cellular and myelin removal, segments were dyed using a standard Luxol fast blue assay with Cresyl violet counterstain. In brief, after deparaffination in Xylenes (Sigma-Aldrich, Cat. No. 247642) for 3 × 2 min, sections were rinsed in 96% ethanol and incubated in 0.1% Luxol Fast Blue solution (CliniSciences, Cat. No. 26056–10) at 56 °C for 20 h. After washing with 96% ethanol samples were differentiated in 0.05% lithium carbonate (Sigma-Aldrich, Cat. No. 62470) and stained with 0.1% cresyl violet acetate solution (Sigma-Aldrich, Cat. No. C5042) for 4 min. The samples were dehydrated and mounted with mounting media [21].

### 2.5. Decellularization Efficiency Analysis

Decellularized tissue was analyzed using immunohistochemistry images. Cross-sections were imaged and the most promising protocols were selected qualitatively. In the following experiments, the decellularization efficiency of selected protocols was confirmed and further optimized. Changes in the decellularization protocols which affected decellularization efficiency negatively were not repeated. However, improvements in the decellularization protocols were repeated and confirmed in the following experiments on nerve tissue from different pigs. This process was repeated for several rounds until one protocol was found that removed immunogenic material and preserved ECM structures. Images were taken using a Nikon Ti2 Eclipse microscope (Nikon, Chiyoda, Tokio, Japan) and CFI Plan Apo Lambda objectives. Fluorescence images were acquired by a photometrics Prime95B camera using a 5-bandpass light filter. Color images were acquired by a Nikon DS-Ri2 camera. 

### 2.6. Quantification of Cellular Components

The decellularization efficiency of the qualitatively selected optimized protocol was quantified. Regions of Interest (ROI) were defined via manual segmentation of fascicles using the NIS-elements software. All subsequent steps were based on the ROI. Autofluorescence of the tissue was subtracted using non-stained negative controls. The remaining MBP, β-tubulin III and DAPI signal after decellularization were then plotted in relation to the signal of unprocessed tissue segments. Images were taken using a Nikon Ti2 Eclipse microscope (Nikon, Chiyoda, Tokio, Japan) and CFI Plan Apo Lambda objectives. Fluorescence images were acquired by a photometrics Prime95B camera using a 5-bandpass light filter. Color images were acquired by a Nikon DS-Ri2 camera. 

### 2.7. Mass Spectrometry-Based Proteome Analysis

#### 2.7.1. Sample Preparation

Three milligrams of sample were lysed in 150 µL of lysis buffer (5% sodium dodecyl sulfate [SDS], 100 mM TEAB, pH = 8) using a PIXUL (Active Motif) sonication device (50 N Pulse, 1 kHz PRF, 20 Hz burst rate). Lysates were centrifuged at 15,000 RCF for 15 min and the protein concentration of the supernatant was assessed using a BCA assay (Thermo Fisher Scientific); 10 µg of protein per sample were reduced by tris (2-carboxyethyl)phosphine at 95 °C for 10 min. Proteins were alkylated using 15 mM iodacetamide at RT in the dark for 30 min and further processed using S-TRAP (Protify) microcartridges, according to the manufacturer’s specifications. For digestion, trypsin was used (1/25 *w*/*w*, ratio trypsin/protein; Promega, Madison, WI, USA) at 47 °C for 1 h. After drying the samples under vacuum, peptides were stored at −20 °C and dissolved in 0.1% aqueous formic acid solution at a concentration of 0.5 mg/mL upon use.

#### 2.7.2. Mass Spectrometry-Based Analysis

For each sample, 0.25 µg total peptides including 5 fmol/µg iRT peptides (Biognosys AG, Schlieren, Switzerland) were subjected to liquid chromatography-mass spectrometry analysis using an Exploris 480 mass spectrometer equipped with a nanoelectrospray ion source (both Thermo Fisher Scientific). Peptide separation was carried out using an Ultimate 3000 System (Thermo Fisher Scientific) equipped with a reverse phase-high performance liquid chromatography column (75 μm × 30 cm) packed in-house with C18 resin (ReproSil-Pur C18-AQ, 1.9 μm resin; Dr. Maisch GmbH, Ammerbuch, Germany) and a custom-made column heater (60 °C). Peptides were separated using a linear gradient from 95% solvent A (0.1% formic acid, 99.9% water) and 5% solvent B (80% acetonitrile, 0.1% formic acid, 19.9% water) to 35% solvent B over 45 min, and further to 50% solvent B over 10 min at a flow rate of 300 nL/min.

For data-independent acquisition (DIA) analysis, each MS1 scan (120k resolution) covering 350 *m/z* to 1400 *m/z* was followed by high collision dissociation (HCD) scans (15k resolution, 22 ms injection time, 8 *m/z* isolation windows with 1 *m/z* overlap, 28 HCD collision energy, 300% automatic gain control) covering the mass range of 400 *m/z* to 900 *m/z*.

#### 2.7.3. DIA Data Analysis 

DIA data was analyzed using Spectronaut (V15.5). A sus scrofa (pig) FASTA was downloaded on 14 October 2021 (unreviewed, one entry per gene). Trypsin/P was set as enzymatic specificity and two missed cleavages were allowed. Oxidation and protein N-terminal acetylation were set as variable modifications, whereas carbamidomethylation (C) was set as a fixed modification. The protein Q value filter was set to 1% for global filtering and to 5% for runwise filtering. Q value filtering was activated, leading to an incomplete protein abundance matrix. 

#### 2.7.4. Data Availability

Raw Data of mass spectrometry experiments are accessible via MassIVE: ftp://MSV000090072@massive.ucsd.edu (accessed on 1 August 2022), username: “MSV000090072_reviewer”, password; “Decellularized”.

### 2.8. hASC Isolation, Characterization, Transplantation and Activity Measures

hASC were isolated and characterized as previously described [22]. Adipose tissue was obtained from healthy human donors undergoing elective liposuction. Informed consent was obtained from the patient prior to liposuction, in addition to approval by the institutional ethics committee of the Basel University Hospital.

In brief, to obtain hASC isolation, fat tissue was cleared from erythrocytes by rinsing with PBS and centrifugation, followed by enzymatic digestion using 0.1% (*w*/*v*) type I collagenase for 3 h at 37 °C and centrifugation at 4 °C for 5 min at 250 g. The resulting cell pellet was resuspended in growth medium (GM) containing Dulbecco’s Modified Eagle’s Medium (DMEM, Gibco-Fisher Scientific, Cat. No. 4196503), 10% fetal bovine serum (FBS), 1% penicillin/streptomycin and 5 ng/mL basic fibroblast growth factor (Peprotech, Rocky Hill, NJ, USA, Cat. No. 100–18B). Cells were cultured for 14 days at 5% CO_2_ and 37 °C. GM was changed every 72 h and cells subdivided at 90% confluency using 0.25% trypsin-EDTA. For cell characterization via flow cytometry, the following fluorophore-conjugated antibodies were used: CD29^+^-PE (Biolegend, Bunkyo Ward, Tokyo, Cat. No. 303003), CD73^+^-APC (Biolegend, Cat. No. 344005), CD90^+^-PE/Cy7 (Biolegend, Cat. No. 328123) and CD105^+^-a488 (Biolegend, San Diego, CA, USA, Cat. No. 323209). Gates were set based on fluorophore-conjugated IgG1 isotype control, namely IgG1-PE (Biolegend, Cat. No. 400111), IgG1-APC (Biolegend, Cat. No. 400119), IgG1-PE/Cy7 (Biolegend, Cat. No. 400125), and IgG1-a488 (Biolegend, Cat. No. 400132). In total, 386,902 CD29^+^CD73^+^CD90^+^CD105^+^ cells were analyzed using a BD FACSAria III (BD Bioscience, Franklin Lakes, NJ, USA). Characterization via Immunocytochemistry was performed as follows. Cells were fixed with 4% PFA for 10 min, then washed with PBS^−/−^. Membrane permeabilization was performed by 0.3% Triton X-100 in PBS^−/−^ for 20 min. Permeabilization solution was washed twice with PBS^−/−^. After 1 h of blocking by 1% protease-free BSA in PBS^−/−^ primary antibodies were added in blocking buffer. Primary antibodies, namely CD105 (Abcam, Cat. No. ab44967), CD44 (Abcam, Cat. No. ab6124), CD90 (Abcam, Cat. No. ab23894) and CD29 (Abcam, Cat. No. ab134179) were incubated overnight at 4 °C in blocking buffer. The remaining primary antibodies were washed away by rinsing cells three times with PBS^−/−^ each time for 10 min. Secondary antibodies (goat anti-rabbit IgG-a546 (Thermo Fisher Scientific, Cat. No. A-11010) and goat anti-mouse IgG-a488 (Thermo Fisher Scientific, Cat. No. A-11029)) and DAPI were added in blocking buffer for 1 h at RT in the dark. Cells were rinsed with PBS^−/−^ and immediately imaged using a Nikon Ti2 Eclipse microscope as previously described (Figure S1).

Decellularized common fibular nerves were cut into 5 mm-long segments and 250k hASC were injected into the grafts using a 30G syringe in a total volume of 50 µL media. Grafts were submerged in GM for a constant supply of nutrients and placed in a 5% CO_2_ incubator at 37 °C. GM contained either 10% FBS as previously described or 5% human platelet lysate (HPL) and 2 U/mL of heparin sodium salt as serum supplement. GM was changed every 48 h. Cell activity was measured by the reduction in resazurin whereby living cells turned from an oxidized blue dye to a pink resorufin product. Cell activity was measured on predefined days 1, 4, 7, 11, 14, 16, 18, 21, 23, 25, 31, 35, 39, 42, 46, 49 and day 53 by a microplate reader, Synergy H1 Hybrid Reader (BioTek, Winooski, VT, USA). Cell activity on day 1 was used for normalization.

### 2.9. Isolation of Embryonic Chicken DRG and SCS

Fertilized chicken eggs were purchased from Gepro Geflügelzucht AG (Flawil, Switzerland). The eggs were incubated for 10 days at 37.8 °C and 100% relative humidity (E10). Embryo collection and dissection were conducted under aseptic conditions in the laminar flow hood with sterile equipment and solutions. For the embryo dissection, a stereomicroscope was utilized. Dissection was performed using previously published protocols for DRG isolation [23,24]. Following DRG isolation, the spinal cord was isolated and segmented into 200 μm-long segments.

### 2.10. Transplantation of DRG and SCS into Acellular Graft

For cell implantation, all the decellularization steps were performed in a sterile environment and the decellularized tissue was treated with 100 U/mL of penicillin, 100 U/mL of streptomycin and 0.25 µg/mL of Gibco amphotericin B for at least 48 h to prevent bacterial and fungal contamination.

Small incisions at the middle of 8 mm-long segments of acellular graft were made using a sterile scalpel. DRG or SCS was implanted into the acellular graft using forceps and held in place using a carrier fibrin hydrogel [22]. The acellular grafts were then placed in a petri dish containing GM as previously described for the hASC culture. GM was replaced every other day. After 6 days, the acellular graft was fixed for 24 h with 4% PFA before embedding in paraffin. Embedded tissue was sectioned in 5 μm thin cross-sections. Implantation of SCS and DRG into the acellular graft was analyzed by staining for β-tubulin III (Figure S2).

## 3. Results

### 3.1. Stepwise Removal of Immunogenic Material from Large-Diameter Pig Nerves

**Figure 2:**
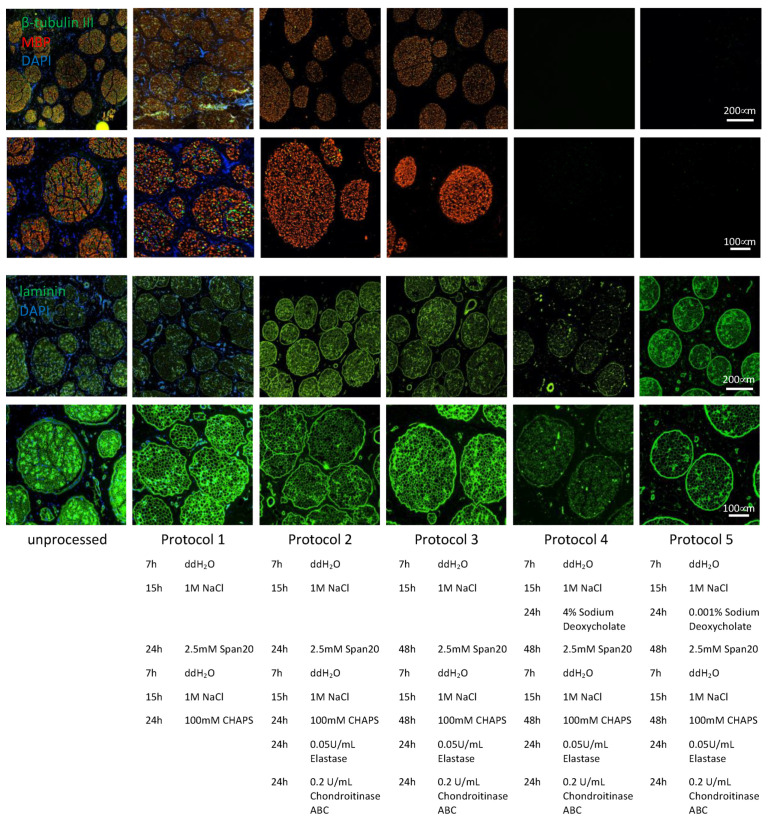
Removal of immunogenic material and ECM preservation by optimized protocols from large diameter pig nerves. First row: scale bar 200 μm, representative images of remaining axonal β-tubulin III, MBP and DAPI. Second row: scale bar 100 μm, zoomed-in representative images of remaining axonal β-tubulin III, MBP and DAPI. Third row: scale bar 200 μm, representative images of remaining ECM laminin structure and DAPI. Fourth row: scale bar 100 μm, zoomed-in representative images of remaining ECM laminin structure and DAPI. Each column shows a further optimization step towards protocol 5, an acellular graft with removed axonal β-tubulin III, MBP, DAPI and a conserved laminin structure signal. Protocol 5 was used for further analysis.

Optimization of the decellularization protocol was performed on 1 cm long sciatic nerve segments. Protocol 1 was previously established for the decellularization of small-diameter rat nerves (data not shown). Applying it to large-diameter sciatic pig nerves resulted in no substantial removal of immunogenic material, such as myelin, axons and DNA. However, also no disruption of the endoneurial tubes was detected after treatment (Figure 2, column 2). Additional enzymatic degradation steps by elastase and chrondroitinase ABC in protocol 2 removed axonal debris and DNA substantially, while preserving the extracellular structure as observed by laminin staining (Figure 2, column 3). Complete removal of axonal debris was achieved by doubling the incubation time of non-ionic Span20 and zwitter-ionic CHAPS from 24 h to 48 h (Figure 2, column 4), but no myelin removal was observed. An additional processing step comprising of the addition of 4% sodium deoxycholate completely removed myelin and any remaining debris. However, the ECM structure was highly disrupted (Figure 2, column 5). Reducing the applied sodium deoxycholate by a factor of 4000× led to the efficient removal of myelin, axons, and DNA, while the ECM structure remained mainly intact and only a minor disruption of the laminin structure was observed (Figure 2, column 6).

### 3.2. Optimized Decellularization Protocol Removes Immunogenic Material in Therapeutically Relevant Nerve Length While Preserving Extracellular Structure

**Figure 3:**
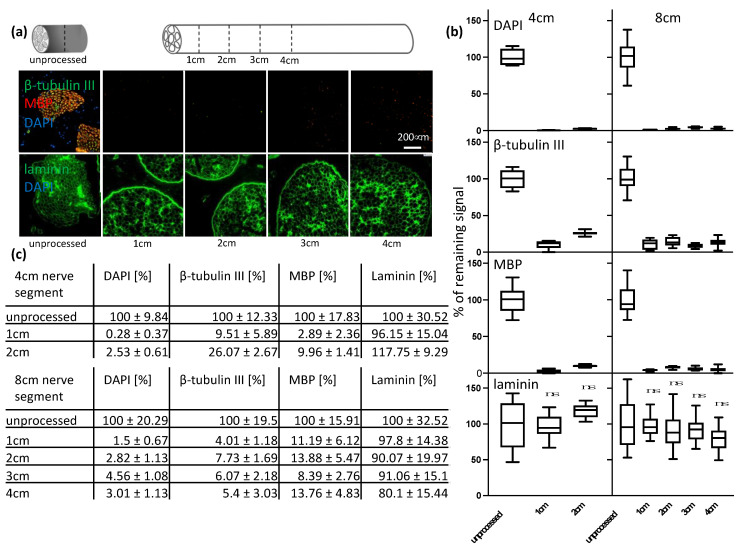
Quantification of remaining cellular components in longer nerve segments. (**a**) The remaining immunogenic material and laminin structure were analyzed every 1 cm up to the middle of an 8 cm-long pig sciatic nerve segment decellularized by protocol 5. Representative images: the remaining ECM laminin structure and DAPI are shown in the upper row, together with the remaining axonal β-tubulin III, MBP and DAPI signals in the bottom row (scale: 200 μm). (**b**) Graphic documentation: the remaining axonal β-tubulin III, MBP, DAPI and laminin signals were quantified. Fascicles were manually segmented and the remaining signals were normalized to signal unprocessed tissue. For statistical analysis, a one-way ANOVA was performed. Adjusted *p*-value: < 0.0001 = ****. ns: no significant differences. (**c**) Numerical documentation: percentage of the remaining axonal β-tubulin III, MBP, DAPI and laminin signals for 4 cm and 8 cm-long segments every 1 cm until the middle.

Sciatic nerves of therapeutically relevant lengths of 4 cm and 8 cm were decellularized using the previously developed protocol 5. As shown in Figure 3a (upper row), immunogenic material can be removed throughout 8 cm-long segments and the intact endoneurial tubes show that the extracellular structure can be well preserved (Figure 3a, lower row). The quantification of the removal of immunogenic material in 4 cm- and 8 cm-long segments also showed a highly significant (adjusted *p*-values < 0.0001) removal of immunogenic material. In addition, no significant removal of laminin was observed (Figure 3b,c). For 4 cm-long segments, after 1 cm only 0.28 ± 0.37% of DAPI, 9.51 ± 5.89% of β-tubulin III and 2.89 ± 2.36% of MBP remained. After 2 cm, in the middle of the 4 cm-long segments, 2.53 ± 0.61% of DAPI, 26.07 ± 2.67% of β-tubulin III and 9.96 ± 1.41% of MBP remained (Figure 3b [left column],c). For 8 cm-long segments, after 1 cm only 1.5 ± 0.67% of DAPI, 4.01 ± 1.18% of β-tubulin III and 11.19 ± 6.12% of MBP remained. After 2 cm, 2.82 ± 1.13% of DAPI, 7.73 ± 1.69% of β-tubulin III and 13.88 ± 5.47% of MBP remained. After 3 cm, 4.56 ± 1.08% of DAPI, 6.07 ± 2.18% of β-tubulin III and 8.39 ± 2.76% of MBP remained. After 4 cm (middle of the 8 cm-long segments), 3.01 ± 1.13% of DAPI, 5.4 ± 3.03% of β-tubulin III and 13.76 ± 4.83% of MBP remained (Figure 3b [right column],c). 

### 3.3. Decellularization Protocol 5 Leads to Preservation of ECM While Removing Lipids and Cellular Content

**Figure 4:**
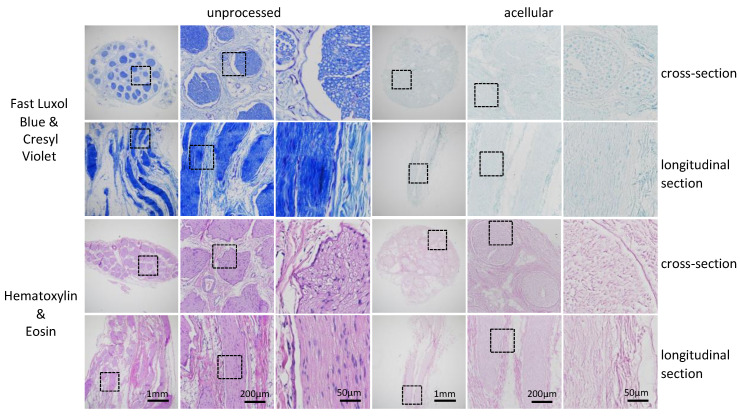
Histological analysis of the ECM and the remaining myelin structure of protocol 5. Luxol fast blue and Cresyl violet stain: myelin (blue) and nuclei (purple) (**top**). Hematoxylin and eosin stain: ECM (pink) and nuclei (purple) (**bottom**). The 5 μm thick cross-sections and longitudinal sections zoomed into framed segments. Scale: 1 mm, 200 μm, 50 μm.

Efficient removal of lipids was confirmed by Luxol fast blue staining of the acellular grafts and showed that dark blue myelin structures within the fascicles were removed completely. Furthermore, it can be observed that the removal of myelin was consistent across the whole diameter and length of the decellularized tissue and no patches of remaining myelin were found. Consistent cell removal was confirmed by the removal of the Cresyl violet stain (purple) (Figure 4, top rows). Preservation of the ECM was shown by hematoxylin and eosin stain (Figure 4, bottom rows). The overall structure of the ECM was well preserved. Although an analysis of higher magnified images indicates a partial disruption of the endoneurial tubes, the ECM matrix was consistently present within the fascicles, but some ring-like structures could still be seen in higher magnified cross-section images of the acellular grafts. In longitudinal sections of hematoxylin and eosin-stained slices, a fibrous structure can be observed in the acellular graft as in unprocessed ones, thus indicating a good cross- and longitudinal preservation of the ECM.

### 3.4. Preservation of the Overall ECM Structure with Minor Disruption of the Endoneurial Tubes

Sciatic nerves of a therapeutically relevant length of 4 cm and 8 cm were decellularized using the newly developed protocol. Immunogenic material could be removed, and the laminin structure was well preserved throughout the large segments as shown previously (Figure 3). However, a minor disruption of the endoneurial tubes could be detected as seen best by hematoxylin and eosin stainings. The ring-like structure of the endoneurial tubes was clearly observable in the magnified hematoxylin and eosin images of the unprocessed tissue, whereas disruption and collapsing of this ring-like structure can be seen in the processed tissue. Additionally, some shrinkage of the tissue and cracks in formation can be observed in processed, but also in unprocessed, segments (Figure 4). 

### 3.5. Decellularization Process Consistently Removes a Significant Proportion of Proteins 

**Figure 5:**
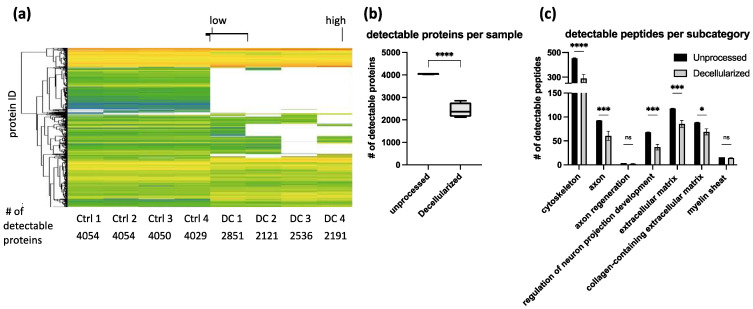
Peptide removal in acellular grafts. Each column represents the protein abundance of one replicate. Ctrl 1–4 are unprocessed samples. DC 1–4 are decellularized samples. (**a**) Heatmap. In total, 4054 proteins were measured. Each line represents one UniProt ID identifying one protein. green: high abundance of protein in the sample. White: low abundancy of protein in the sample. (**b**) Total number of proteins above detection limit. (**c**) Number of peptides per subcategory. Subcategories defined by AmiGO2 GO class for *sus scrofa* and UniPort as contributor. The bar plot shows the number of detectable peptides in all replicates in chosen subcategories (cytoskeleton, axon-, axon regeneration, regulation of neuron projection development-, ECM-, collagen-containing ECM, myelin sheath associated peptides). For statistical analysis, Two-way ANOVA was performed. Adjusted *p*-value: <0.0001 = ****, *p* < 0.001 = ***, *p* < 0.01 = **, *p* < 0.05 = * ns: no significant differences.

Using mass spectrometry, an average 4046 ± 10 proteins could be detected in unprocessed tissue, whereas in decellularized tissue, an average of 2424 ± 292 proteins were detected (Figure 5b). In total, 1078 proteins were no longer detectable in any of the acellular samples. This list of proteins no longer detectable after decellularization contain known inhibitors to axonal regeneration such as reticulon, also known as Nogo, and chondroitin sulfate proteoglycan 4, which was specifically enzymatically removed using chondroitinase ABC during the decellularization process (Table S1). By categorizing detected peptides into subcategories, it can be seen that several peptides from all chosen subcategories are no longer detectable in acellular grafts. Cytoskeleton-associated peptides were significantly reduced from 454 ± 2.7 in unprocessed grafts to 289 ± 28.7 in acellular grafts. The number of peptides associated with axons was significantly reduced from 93 ± 0.4 peptides in unprocessed grafts to 60.8 ± 8.3 in acellular grafts. The 4 ± 0 axon regeneration-associated peptides were detected in unprocessed grafts versus 2.5 ± 0.5 in acellular grafts. Regulation of neuron projection development-associated peptides were significantly reduced from 68.3 ± 0.4 to 37 ± 5.1. ECM-associated peptides were significantly reduced from 117.8 ± 0.4 to 85.5 ± 6.3. Collagen-containing ECM-associated peptides were significantly reduced from 88.8 ± 0.4 to 69 ± 5.6. Finally, 16 ± 0 peptides associated with the myelin sheath were above the detection limit in unprocessed grafts whereas 14.5 ± 0.5 were detectable after decellularization (Figure 5c). 

### 3.6. Decellularized Porcine Sciatic Nerves Suitable for Human Cell Transplantation

**Figure 6:**
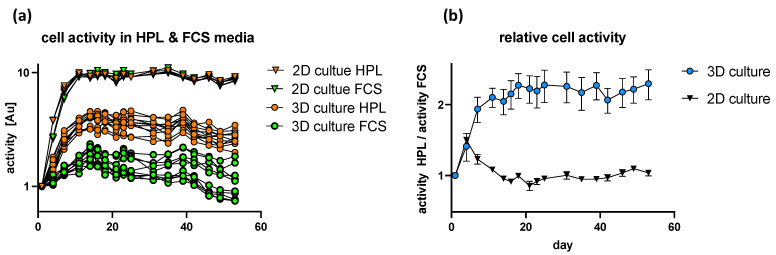
Decellularized nerve as 3D-scaffold for hASC culture. (**a**) 250k hASC were injected per 3D acellular graft (dots), 10k hASC seeded in 2D-culture well as a control (triangles). Cellular activity was analyzed using resazurin every 3–5 days in media supplemented by 10% FCS (green) or 5% HPL and 2 U/mL heparin (orange). Cell activity normalized to cell activity at day 1 post-transplantation. Triangles represent cell activity in 2D-culture plates and dots represent cell activity in 3D-culture. (**b**) cell activity regarding FCS- and HPL-supplemented media. Relative cellular activity of hASC cultured in HPL-supplemented media normalized to relative cellular activity of hASC cultured in FCS-supplemented media. Black triangles represent normalized cell activity in 2D culture and blue dots represent normalized cell activity in 3D.

Isolated hASC were analyzed using flow cytometry; 81% of cells were CD29+CD73+CD90+CD105+ (Figure S1a). These findings were confirmed via immunocytochemistry. Additionally, expression of the MSC marker CD44 was shown via immunocytochemistry stainings (Figure S1b). hASC were injected into decellularized porcine sciatic nerves either in fetal calf serum (FCS) or HPL-supplemented GM. Heparin was added in the latter case to avoid clocking. Cell activity increased within the first 11 days in standard 2D culture and remained constantly high, regardless of the used media. An increase in cellular activity could be observed along 14 days post-transplantation into the graft using HPL-containing media only. In media containing FCS, cellular activity remained constant over the course of the experiment and did not increase or vanish, thus indicating cell survival, but lack of proliferation. The absolute cellular activity of cells cultured in the nerve scaffolds remained below the cellular activity of 2D-cultured cells, regardless of the chosen media (Figure 6a). However, comparing cell activity in 2D- vs. A 3D culture is error-prone due to different growth area/volumes in which cells can grow. Comparing the effect of FCS- versus HPL-containing media did not show any relative difference when cells were cultured in a 2D system. However, when comparing HPL- to FCS-containing media in the nerve scaffolds, a 2.2-fold increase in relative cell activity could be observed within the first 14 days and remained constant until termination of the experiment at day 53 (Figure 6b). Further, acellular grafts can be used as a 3D culture system for embryonic chicken tissue explants as seen by DRGs and SCS which were cultured for up to 6 days in the acellular grafts. DRGs and SCS can be detected over a distance up to 500 μm by axonal staining (Figure S2).

## 4. Discussion

The objective of this study was to develop and establish a protocol for the efficient removal of immunogenic material from large diameter/longer peripheral nerve segments with only a simultaneous minimal impact on the ECM structure of the nerve. For potential future clinical applications, domestic pigs were chosen as the donor source due to their comparable size to humans. Our study shows that these objectives were achieved by combining various zwitterionic, non-ionic and ionic detergents together with enzymatic treatments. Furthermore, it could be demonstrated that after the decellularization process, the grafts did not show cytotoxicity as demonstrated by human cell studies. 

Adapting the protocol previously used for the decellularization of rat sciatic nerve, i.e., 24 h of zwitterionic CHAPS treatment as described by Hudson in 2004, followed by 24 h of treatment using the non-ionic surfactant Span20 required several optimization steps as shown in Figure 2 [16]. Due to the large size and increased amount of connective tissue of porcine sciatic nerves compared to adult rat sciatic nerves, the previous protocol was not efficient in removing immunogenic material such as DNA, axons and myelin (Figure 2, protocol 1). As previously described by Hundepool et al. in 2017, adding enzymatic treatment, such as elastase, helped to remove axons and Schwann cells while preserving the ECM structure [20]. In addition, to improve nerve regeneration, chondroitin sulfate proteoglycans, which inhibit axonal regeneration, were enzymatically degraded using chondroitinase ABC as described by Im et al. in 2019 [25]. Chondroitin sulfate proteoglycan 4 is the only detectable chondroitin sulfate proteoglycan in the unprocessed samples and is completely removed in all four DC samples (Table S1). These enzymatic treatments allowed us to remove the vast majority of axonal debris and DNA without disrupting the ECM structure (Figure 2, protocol 2). To remove the last parts of axonal debris and DNA, the incubation times for CHAPS and Span20 were doubled from 24 h to 48 h to allow the detergents to disperse throughout the several-fold larger segments of pig nerve. By this, axons and DNA were removed entirely from the tissue, but myelin removal was not accomplished with this protocol (Figure 2, protocol 3).

As described in 1983 [26], sodium deoxycholate can be used for the solubilization and extraction of myelin from nerve tissue. As recently reported, sodium deoxycholate has also been used in decellularization [27]. Nevertheless, due to the relatively large size of the tissue and to allow complete perfusion of the detergents, we did not want to reduce treatment duration. With a concentration described previously for the solubilization of myelin and decellularization, myelin removal was highly efficient, and it was no longer detectable after 24 h of treatment. However, the protein denaturing capacities of the anionic sodium deoxycholate disrupted the extracellular structure as indicated by disruption of the laminin structure. After treatment with 4% sodium deoxycholate, the laminin structure found in unprocessed tissue of the endo- pero- and epi-neurium was highly segmented (Figure 2, protocol 4). To reduce the impact on the extracellular structure while maintaining efficient myelin removal, sodium deoxycholate was titrated down from an initial concentration of 40 g/L to a final concentration of 10 mg/L. In this way, myelin removal was still achieved, and undesired ECM damage was significantly reduced (Figure 2, protocol 5).

Thus, the goal of this study was to develop a decellularization protocol for a clinically relevant size of nerve segments. Once an efficient decellularization protocol was established for 1 cm-long segments of the porcine sciatic nerve without any disruption of the ECM structure; further, longer segments of 4 cm and 8 cm were decellularized. It was assumed that given the increased size of the tissue, the active agents would need more time to penetrate the tissue and wash out debris. However, we were able to show that the efficient removal of immunogenic material was successful throughout longer segments. Following signal quantification, we observed residual signal of axonal and myelin signals, but little for DNA (Figure 3). We concluded that the consequence of the further removal of immunogenic material would result in a major disruption of the ECM structure. To confirm our findings, we analyzed the tissue by immunohistochemistry. A previous analysis conducted using antibody staining led to uncertainty. If no positive signal can be detected any longer, we cannot be certain if the extensive decellularization process has led to a removal of the whole protein as targeted or to a disruption of the epitope only. Therefore, by using immunohistochemistry, namely hematoxylin and eosin, as well as Luxol fast blue and Creysl violet staining, we were able to confirm what we observed using immunocytochemistry (Figure 4). Additionally, by the broad staining of the histological stains, we can exclude that the newly developed decellularization protocol acts on single proteins only and that an overall removal of cytoplasmic proteins, nuclei and lipids can be achieved, as well as a preservation of the overall ECM structure and not only laminin. However, it can be observed by laminin staining of the processed tissue that the multilayered perineurium structure is loosened. We hypothesize that this effect is the result of the enzymatic degradation of the elastin within the perineurium by elastase, which is necessary to achieve efficient axonal removal (Figure 3). As tissue shrinkage and crack formation was observed in processed and in unprocessed tissue, we conclude that this arises due to the standard dehydration steps required for paraffin embedding of the tissue and not due to the decellularization process itself (Figure 4). In general, we conclude that the remaining nerve structure should be sufficiently well preserved to act as a guiding structure for elongating axons, blood vessels and cell distribution across the grafts as it resembles the native ECM structure in much greater detail than any of the artificial biological and non-biological nerve guiding conduits used for nerve regeneration applications [28,29,30,31,32].

Peptide removal among repeated decellularization experiments was consistent as evidenced by proteomics analysis (Figure 5). In our optimized protocol, around 1500 proteins were reduced below the detection limit. Categorizing them into clusters of interest showed that peptide removal was not specific to certain categories. Peptides were considered as removed when each of the four decellularized replicates was below the technical detection limit. Proteins that are strongly removed, but still detectable, were not listed as removed (Table S1). Quantitative conclusions about the amount of remaining protein are limited due to the lack of appropriate controls for tissue loss during the decellularization process. The strong impact of the decellularization process on protein content renders impossible a normalization approach to directly compare the number of single proteins to each other in unprocessed and acellular grafts (Figure 5). For proteomics analysis, only 3 mg of tissue was used per sample. To lower the variability among the samples, complete acellular grafts should be homogenized. By this, artifacts in sample collection could be reduced and variability lowered.

As expected, proteomics analysis revealed partial removal of ECM-associated peptides. However, IHC images confirm the preservation of the overall ECM structure as previously discussed which is crucial as a guiding structure for regenerating axons. Using IHC, laminin was analyzed specifically due to its biological relevance. It has been shown that laminin is crucial for nerve regeneration and remyelination by Schwann cells [33]. Therefore, it is of greatest importance to preserve the laminin structure during decellularization as shown by IHC. Even though direct proof of successful nerve regeneration using our acellular conduits is lacking, efficient nerve reinnervation due to the observed in vitro results can be expected. 

Based on previous studies, it could be shown that HPL-supplemented media significantly improved the neurotrophic potency of hASC as shown by robust axonal outgrowth in vitro [34]. Interestingly we could show that serum supplements have a different impact on cells being cultured in 2D culture or in 3D acellular nerve scaffolds. Whereas cellular activity increased in a comparable manner in 2D culture, regardless of the chosen media composition, HPL-supplemented media had a positive impact on cellular activity in 3D nerve scaffolds, whereas FCS-supplemented media did not induce a rise in cellular activity. We conclude that the experimental design is not sensitive enough at beginning of the experiments for the 2D culture system. Based on the literature, we expected a positive effect of HPL on cell activity in a 2D culture system as well [35]. To increase experimental sensitivity, the starting cell density has to be lowered and cell activity measured more regularly. Combining these findings with the improved neurotrophic potency of hASC by HPL-supplemented media gives us a viable system for future studies of axonal outgrowth and nerve regeneration both in vitro, as well as in vivo. Additionally, the first proof of biocompatibility was shown by the implantation of human tissue-derived mesenchymal stem cells. Therefore, we can conclude that potentially harmful factors of the decellularization process were sufficiently removed and that the graft could be used for in vivo nerve regeneration studies (Figure 6). According to the 3R principle of animal experimentation, we were able to show that by extensive in vitro analysis of the remaining factors and structural integrity by immunocytochemistry, immunohistochemistry, proteomics analysis, and biocompatibility by primary human cell implantation, new functional decellularization protocols can be established without the need of animal studies.

During this study, primary experiments were conducted for the implantation of DRG and SCS of chicken embryos to study axonal outgrowth in the acellular graft and to examine the usability of the grafts as a 3D culture system. For this, transplants were placed in a small incision in the graft and held in place by a fibrin hydrogel carrier. Following incubation, fixation, paraffin embedding and sectioning, immunocytochemistry images were taken as described previously in the methods. Based on that, it was expected to detect axonal elongation and compare the axonal elongation rate as an in vitro model of axonal regeneration. DAPI and β-tubulin III signaling was clearly detectable at the place of implantation. However, the achieved resolution and the uncertainty of the exact graft implantation site did not allow any conclusion about the axonal signal outside the implant or about axonal elongation. Therefore, no conclusion about the axonal elongation rate can be made (Figure S2). Nevertheless, we can conclude that DRG and SCS implantation can be performed using this experimental setup and that they can survive for several days within the acellular grafts. Of note, quantification of the amount of axonal elongation and an improved analysis adapted to a 3D system is necessary. By this, we expect that the acellular 3D grafts can be used to study axonal outgrowth as previously shown in a 2D in vitro setup [34].

We are aware that the lack of in vivo animal studies is a limitation of our work and would allow a more direct conclusion about biocompatibility, particularly axonal outgrowth and the regain of motor and sensory function and behavior outcome. However, the need for large animals required for large nerve gap studies with human nerve-sized implants and the resulting costs and ethical concerns makes it even more important for newly developed decellularization protocols to be improved and tested extensively in vitro before translating to in vivo studies. 

## 5. Conclusions

Our study reports a protocol for efficient decellularization of larger segments of porcine peripheral nerves. It was shown that DC protocols established in a rodent model require substantial adaptions for being efficient in large segments of porcine nerves. Based on extended in vitro optimization and testing, a protocol was developed which allows immunogenic material removal while preserving the overall nerve structure. Proteomic analysis and cell culture experiments indicate biocompatibility of the acellular graft and suitability for application in transplantation studies. Furthermore, recellularization of acellular porcine nerve grafts with autologous hASC and autologous HPL may open a new option for in vitro generation of a functional, non-immunogenic personalized nerve graft circumventing the major drawbacks associated with autologous nerve grafting.

## Data Availability

Raw Data of mass spectrometry experiments are accessible via MassIVE: ftp://MSV000090072@massive.ucsd.edu (accessed on 1 August 2022), username: “MSV000090072_reviewer”, password; “Decellularized”.

## Supplementary Materials

The following supporting information can be downloaded at: https://www.mdpi.com/article/10.3390/bioengineering9090412/s1, Figure S1: hASC characterization; Figure S2: chicken Spinal Cord Segment and Dorsal Root Ganglion transplantation; Table S1: proteins removed below detection limit by decellularization in all replicates.
