# Supplementary material for: Optimized Decellularization Protocol for Large Peripheral Nerve Segments: Towards Personalized Nerve Bioengineering
Source: Bioengineering (Basel). 2022 Aug 24;9(9):412. doi: 10.3390/bioengineering9090412 (PMC9495622; doi:10.3390/bioengineering9090412)
Supplement: Supplementary file 1 [file bioengineering-09-00412-s001.zip › bioengineering-1872486-supplementary.pdf]

bioengineering

MDPI

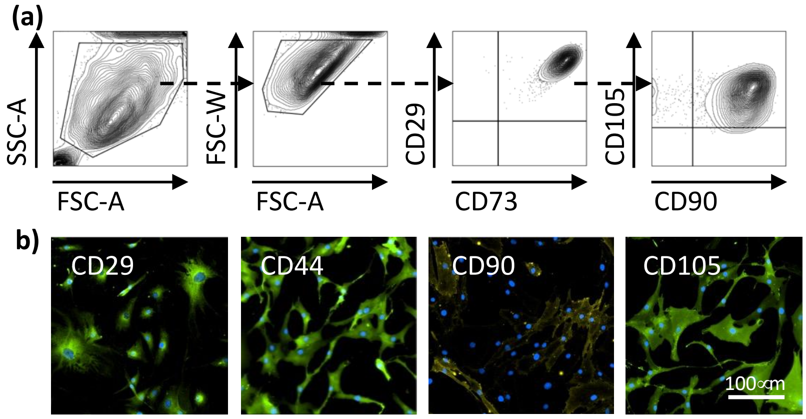

Supplementary Figure S1: hASC characterization. **a)** flow cytometry characterization of hASC. Gates were set according to IgG negative controls. **b)** Immunocytochemistry of hASC. Scale: 100 μm.

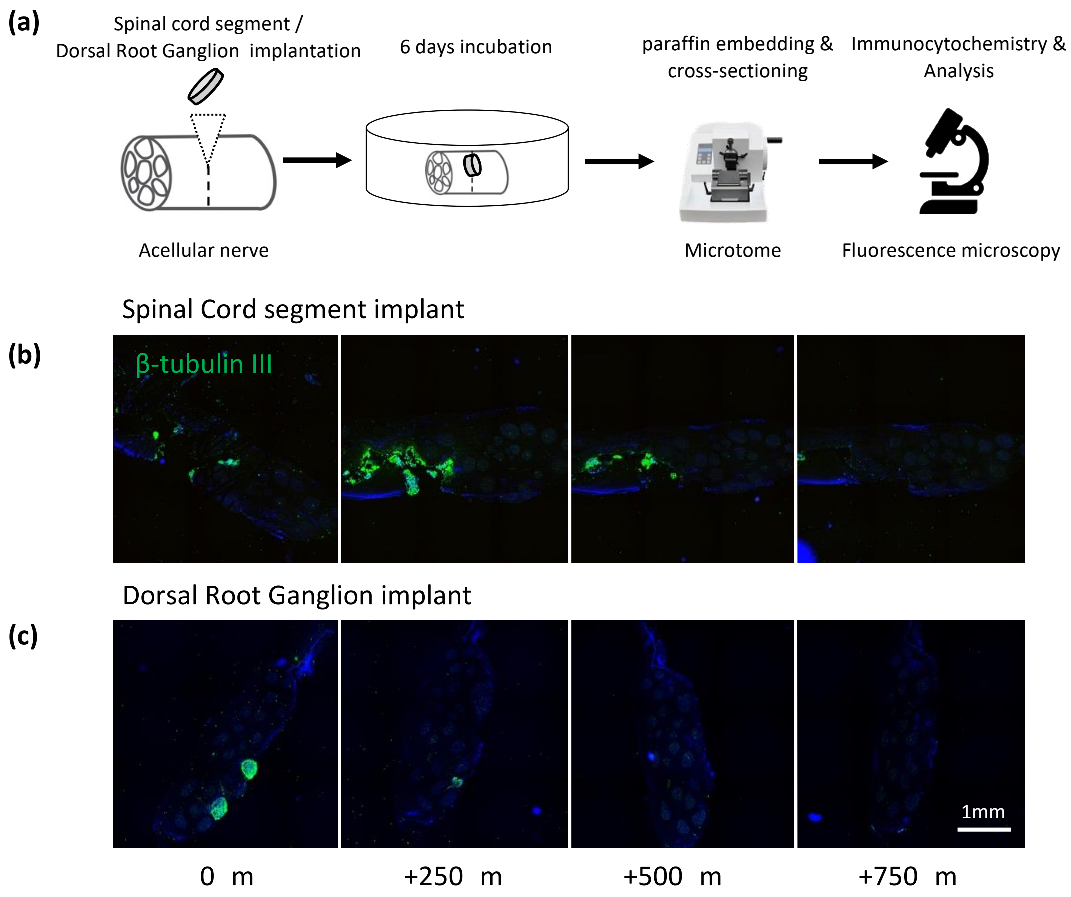

Supplementary Figure S2: chicken Spinal Cord Segment and Dorsal Root Ganglion transplantation. **a)** experimental setup. Spinal Cord and Dorsal root ganglion were isolated from chicken embryo at day 11 post-fertilization. Tissue was implanted in a incision in the graft and hold in place by a drop of methylcellulose. **b)** cross-sections of acellular graft with spinal cord segments implants. **c)** cross-sections of acellular graft with Dorsal root ganglion implants. Images were taken every 250μm of the graft across the whole β-tubulin III signal. 0μm indicated the beginning of observed signal, not site of implantation. scale: 1mm

**Supplementary Table S1:** proteins below detection limit in all acellular replicates.
1-acylglycerol-3-phosphate O-acyltransferase ABHD5
2-methoxy-6-polyprenyl-1,4-benzoquinol methylase, mitochondrial
2,4-dienoyl-CoA reductase 2
2'-5'-oligoadenylate synthase 1
26S proteasome non-ATPase regulatory subunit 9
26S proteasome regulatory subunit 8
28S ribosomal protein S36, mitochondrial
3-hydroxy-3-methylglutaryl coenzyme A reductase;3-hydroxy-3-methylglutaryl coenzyme A reductase;3-hydroxy-3-methylglutaryl-coenzyme A reductase
3-hydroxybutyrate dehydrogenase 1
3-hydroxyisobutyrate dehydrogenase
39S ribosomal protein L16, mitochondrial
39S ribosomal protein L17, mitochondrial
39S ribosomal protein L38, mitochondrial
4-hydroxy-2-oxoglutarate aldolase 1;4-hydroxy-2-oxoglutarate aldolase, mitochondrial
40S ribosomal protein S30
4HBT domain-containing protein
5'-nucleotidase domain containing 2
5'-nucleotidase domain containing 3
5'-nucleotidase, cytosolic II;5'-nucleotidase, cytosolic II;5'-nucleotidase, cytosolic II;5'-nucleotidase, cytosolic II;5'-nucleotidase, cytosolic II;5'-nucleotidase, cytosolic II
60S ribosomal protein L26-like 1 isoform X1
72 kDa gelatinase
A-kinase anchoring protein 6;A-kinase anchor protein 6
AAA domain-containing protein
Abhydrolase domain containing 11
Abhydrolase domain containing 4, N-acyl phospholipase B;Abhydrolase domain containing 4, N-acyl phospholipase B;Abhydrolase domain containing 4, N-acyl phospholipase B;Abhydrolase domain containing 4, N-acyl phospholipase B;Abhydrolase domain containing 4, N-acyl phospholipase B;Abhydrolase domain containing 4, N-acyl phospholipase B
Acetyl-CoA C-myristoyltransferase
Acidic fibroblast growth factor intracellular-binding protein isoform b
Actin binding LIM protein family member 3
Actin-depolymerizing factor
Active breakpoint cluster region-related protein
Acyl-CoA dehydrogenase family member 10
Acyl-CoA synthetase family member 3
Acyl-CoA synthetase long chain family member 4
Acyl-CoA synthetase long chain family member 6
Acyl-CoA thioesterase 8

Acyl-CoA-binding domain-containing protein 5;Acyl-CoA-binding domain-containing protein 5;Acyl-CoA-binding domain-containing protein 5;Acyl-CoA binding domain containing 5;Acyl-CoA-binding domain-containing protein 5
ADAM metallopeptidase domain 23
ADAMTS like 1
ADAMTS like 3
ADAMTS like 4
ADAMTS like 5
Adducin 1
Adenosine aminohydrolase;Adenosine deaminase;Adenosine deaminase;Adenosine deaminase
Adenosine deaminase RNA specific
Adenylate kinase 4, mitochondrial
ADP ribosylation factor GTPase activating protein 1
ADP ribosylation factor interacting protein 1
ADP ribosylation factor like GTPase 10
ADP-ribosylation factor-related protein 1 isoform X1;Uncharacterized protein
Afadin, adherens junction formation factor;Afadin, adherens junction formation factor;Afadin, adherens junction formation factor;Afadin, adherens junction formation factor;Afadin, adherens junction formation factor;Afadin, adherens junction formation factor;Afadin, adherens junction formation factor
Afamin
Agenet-like domain-containing protein;Agenet-like domain-containing protein;Uncharacterized protein
AHNAK nucleoprotein
AIG1-type G domain-containing protein
Alcohol dehydrogenase iron-containing protein 1
Alpha-(1,6)-fucosyltransferase;Alpha-(1,6)-fucosyltransferase;Alpha-(1,6)-fucosyltransferase
alpha-1,2-Mannosidase;alpha-1,2-Mannosidase
Alpha-1,3-mannosyl-glycoprotein 2-beta-N-acetylglucosaminyltransferase
Alpha-1,3/1,6-mannosyltransferase ALG2
Alpha-2-glycoprotein 1, zinc-binding;Alpha-2-glycoprotein 1, zinc-binding;Alpha-2-glycoprotein 1, zinc-binding
Alpha-2-macroglobulin receptor-associated protein
Alpha-mannosidase
Alpha-tubulin N-acetyltransferase 1
Amine oxidase
Amino_oxidase domain-containing protein;Uncharacterized protein
Aminoacyl-tRNA hydrolase
Amyloid-beta A4 protein
Angiopoietin like 2
Angiopoietin like 7
Ankyrin repeat and MYND domain containing 2
Ankyrin repeat and sterile alpha motif domain containing 3;Ankyrin repeat and sterile alpha motif domain containing 3;Ankyrin repeat and sterile alpha motif domain containing 3;Ankyrin repeat and SAM domain-containing protein 3 isoform 1
Ankyrin repeat domain 27

Ankyrin repeat domain 29
Anoctamin
ANTXR cell adhesion molecule 1
AP complex subunit sigma
APC-binding protein EB1
Aph-1 homolog A, gamma-secretase subunit
Apolipoprotein A-IV
Apolipoprotein C-III
Apolipoprotein H
Apoptosis inhibitor 5
Apoptotic chromatin condensation inducer 1
Arf-GAP with SH3 domain, ANK repeat and PH domain-containing protein 2 isoform a;Arf-GAP with SH3 domain, ANK repeat and PH domain-containing protein 2 isoform X2;ArfGAP with SH3 domain, ankyrin repeat and PH domain 2;ArfGAP with SH3 domain, ankyrin repeat and PH domain 2;ArfGAP with SH3 domain, ankyrin repeat and PH domain 2
ArfGAP with GTPase domain, ankyrin repeat and PH domain 3;Arf-GAP with GTPase, ANK repeat and PH domain-containing protein 3 isoform a;Arf-GAP with GTPase, ANK repeat and PH domain-containing protein 3 isoform a;ArfGAP with GTPase domain, ankyrin repeat and PH domain 3;ArfGAP with GTPase domain, ankyrin repeat and PH domain 3;ArfGAP with GTPase domain, ankyrin repeat and PH domain 3;ArfGAP with GTPase domain, ankyrin repeat and PH domain 3
ARFGEF family member 3
Arginyl-tRNA synthetase
Armadillo like helical domain containing 3
Arsenite-resistance protein 2
Arylsulfatase B
Asc-type amino acid transporter 1
Asparagine synthetase [glutamine-hydrolyzing]
Asparaginyl endopeptidase;Legumain;Legumain;Asparaginyl endopeptidase;Asparaginyl endopeptidase
Aspartyl-tRNA synthetase 2, mitochondrial;Aspartyl-tRNA synthetase 2, mitochondrial
ATP binding cassette subfamily B member 8
ATP binding cassette subfamily C member 8
ATP binding cassette subfamily D member 1
ATP synthase subunit d, mitochondrial;ATP synthase subunit d, mitochondrial;ATP synthase subunit d, mitochondrial
ATP synthase subunit s, mitochondrial;ATP synthase subunit s, mitochondrial
ATP-dependent RNA helicase DHX29
ATP/GTP binding protein 1
Atypical kinase COQ8A, mitochondrial;Atypical kinase COQ8A, mitochondrial;Atypical kinase COQ8A, mitochondrial;Atypical kinase COQ8A, mitochondrial;Atypical kinase COQ8A, mitochondrial
Autophagy-related protein 101
Axonal membrane protein GAP-43;Growth associated protein 43;Axonal membrane protein GAP-43
Band 4.1-like protein 2 isoform a
BCAS3 microtubule associated cell migration factor
BCL2 associated transcription factor 1

BCL2 like 13
Beta_elim_lyase domain-containing protein
Beta-1,4-glucuronyltransferase 1
Beta-2-microglobulin
Beta-ketoacyl-[acyl-carrier-protein] synthase I;3-oxoacyl-[acyl-carrier-protein] synthase, mitochondrial
Beta-MPP;Peptidase, mitochondrial processing subunit beta
BICD cargo adaptor 2
Bifunctional polynucleotide phosphatase/kinase;Polynucleotide kinase 3'-phosphatase;Polynucleotide kinase 3'-phosphatase;Polynucleotide kinase 3'-phosphatase;Polynucleotide kinase 3'-phosphatase;Polynucleotide kinase 3'-phosphatase
BR serine/threonine kinase 1;BR serine/threonine kinase 1;BR serine/threonine kinase 2;BR serine/threonine kinase 2;BR serine/threonine kinase 1;BR serine/threonine kinase 2;BR serine/threonine kinase 2
Branched-chain-amino-acid aminotransferase
BRISC and BRCA1-A complex member 1
Brix domain-containing protein 2
Bromodomain adjacent to zinc finger domain 1B
BTB/POZ domain-containing protein KCTD4
C-C motif chemokine
C-type lectin domain containing 11A
C1q domain-containing protein
C1q domain-containing protein;Complement C1q and tumor necrosis factor-related protein 9A
C2CD2 like
C3/C5 convertase
Cadherin 11
Calcium binding and coiled-coil domain 1
Calcium signal-modulating cyclophilin ligand
Calcium uptake protein 2, mitochondrial;Mitochondrial calcium uptake 2;Mitochondrial calcium uptake 2
Calcium-binding mitochondrial carrier protein SCaMC-3;Solute carrier family 25 member 23;Small calcium-binding mitochondrial carrier 3;Solute carrier family 25 member 23
Calcium-transporting ATPase
Calcium/calmodulin dependent protein kinase I
Calcium/calmodulin dependent protein kinase ID
Calcium/calmodulin dependent protein kinase kinase 1
Calmin
Calpain 6
Calponin
Calsyntenin 1
Canopy FGF signaling regulator 4
CAP-ZIP_m domain-containing protein
Carbonyl reductase 4
Carboxylic ester hydrolase
Carboxypeptidase
Carboxypeptidase X, M14 family member 1

CASK interacting protein 2
Caspase 7
Caspase recruitment domain family member 19
Catechol O-methyltransferase domain-containing protein 1
Catenin beta like 1
CCN family member 2
CD248 molecule
CD302 antigen
CD74 molecule;CD74 molecule;CD74 molecule;CD74 molecule;CD74 antigen
CDC42 effector protein 1
CDK5 regulatory subunit associated protein 3
CDP-diacylglycerol synthase;Phosphatidate cytidylyltransferase, mitochondrial;Phosphatidate cytidylyltransferase, mitochondrial;CDP-diacylglycerol synthase
Cell adhesion molecule 1
Cell adhesion molecule 2
Cell cycle associated protein 1
Cell division cycle 5 like
Cell division cycle protein 123 homolog
Centrosomal protein 250
Cerebellar degeneration related protein 2 like;Cerebellar degeneration-related protein 2-like
Chemerin
Chloride channel protein
Chondroitin sulfate proteoglycan 4
Chondromodulin
Christmas factor
Chromatin target of PRMT1;Chromatin target of PRMT1;Chromatin target of PRMT1;C1orf77 protein
Chromobox 5
Chromobox protein homolog 3 isoform X1;Uncharacterized protein
Chromosome 1 C15orf48 homolog
Chromosome 16 C5orf51 homolog
Citramalyl-CoA lyase
Clathrin light chain
Cleavage and polyadenylation specific factor 3
Cleavage and polyadenylation specific factor 7
Cleavage and polyadenylation specificity factor subunit 6
Cleavage stimulation factor subunit 3
Coactivator-associated arginine methyltransferase 1
Coagulation factor III;Tissue factor
Coagulation factor XII
Coatomer subunit zeta
Coiled-coil domain containing 91
Coiled-coil domain-containing protein 128

Coiled-coil domain-containing protein 47
Coiled-coil domain-containing protein 93
Coiled-coil serine rich protein 2
Coiled-coil-helix-coiled-coil-helix domain containing 6
Coilin
COMM domain containing 10
COMM domain-containing protein 5
Complement component 1 Q subcomponent-binding protein, mitochondrial;Complement component 1 Q subcomponent-binding protein, mitochondrial
Complex I subunit B13
Complex I-15 kDa
Complex I-20kD;Complex I-20kD;NADH:ubiquinone oxidoreductase core subunit S7;Complex I-20kD;Complex I-20kD
Complex I-23kD
Complex I-9kD
Complex I-B14
Complex I-B15
Complex I-B18
Complex I-ESSS
Complex III subunit 8
COP9 signalosome complex subunit 4
Copine 4
Copper chaperone for superoxide dismutase;Superoxide dismutase [Cu-Zn]
Coproporphyrinogen oxidase
Core histone macro-H2A
Coronin
Corrinoid adenosyltransferase
CortBP2 domain-containing protein;CortBP2 domain-containing protein;Cortactin-binding protein 2;Cortactin-binding protein 2
Crystallin beta-gamma domain containing 3
CUGBP Elav-like family member 2
CWC22 spliceosome associated protein homolog
Cyclin G associated kinase
Cysteine and histidine-rich domain-containing protein 1
Cysteine rich secretory protein LCCL domain containing 2
Cysteinyl-tRNA synthetase
Cysteinyl-tRNA synthetase;Cysteinyl-tRNA synthetase;Cysteinyl-tRNA synthetase 2, mitochondrial;Cysteinyl-tRNA synthetase;Cysteinyl-tRNA synthetase 2, mitochondrial
Cytidine/uridine monophosphate kinase 2
Cytochorme P450 2A19
Cytochrome b reductase 1
Cytochrome b-c1 complex subunit 10
Cytochrome b-c1 complex subunit 6

Cytochrome c domain-containing protein
Cytochrome c oxidase assembly homolog COX15
Cytochrome c oxidase subunit
Cytochrome c oxidase subunit 4
Cytochrome c oxidase subunit 7A2 like
Cytochrome P450 1B1
Cytoplasmic linker associated protein 2
Cytoplasmic protein
Cytosolic arginine sensor for mTORC1 subunit 1
Cytosolic Fe-S cluster assembly factor NUBP2
Cytospin-A
D-aminoacyl-tRNA deacylase
DAB adaptor protein 2
DDB1 and CUL4 associated factor 1
DDB1 and CUL4 associated factor 7
DDRGK domain-containing protein 1
Dehydrogenase/reductase SDR family member on chromosome X;Uncharacterized protein
Dematin actin binding protein
DENN domain containing 5A
Density-regulated protein
Deoxynucleoside triphosphate triphosphohydrolase SAMHD1
DEP domain-containing mTOR-interacting protein isoform 1;Uncharacterized protein;Uncharacterized protein
DFRP_C domain-containing protein;RWD domain-containing protein
Diacylglycerol kinase
Diaphanous related formin 1
Dimethylaniline monooxygenase [N-oxide-forming]
Dishevelled associated activator of morphogenesis 1;Disheveled-associated activator of morphogenesis 1 isoform 2;Dishevelled associated activator of morphogenesis 1
DM1 locus, WD repeat containing;DM1 locus, WD repeat containing
Dmx like 1
Dmx like 2
DNA helicase
DNA polymerase delta interacting protein 2
DNA polymerase delta interacting protein 3
DNA-directed RNA polymerase II subunit RPB3
DnaJ heat shock protein family (Hsp40) member B4
DnaJ homolog subfamily C member 10
Dolichyl-diphosphooligosaccharide--protein glycosyltransferase subunit KCP2
Dolichyl-phosphate beta-glucosyltransferase
Dolichyl-phosphate-mannose--protein mannosyltransferase
Dopamine beta-hydroxylase

Doublecortin like kinase 2
Down syndrome critical region protein 3 isoform 1;VPS26 endosomal protein sorting factor C;VPS26 endosomal protein sorting factor C
dTMP kinase
Dual specificity mitogen-activated protein kinase kinase 7 isoform 1;Mitogen-activated protein kinase kinase 7
Dynamin-like 120 kDa protein, form S1;Dynamin-like 120 kDa protein, form S1
Dystrophin
E3 ubiquitin-protein ligase
E3 ubiquitin-protein ligase HACE1 isoform b;HECT domain and ankyrin repeat containing E3 ubiquitin protein ligase 1;HECT domain and ankyrin repeat containing E3 ubiquitin protein ligase 1;E3 ubiquitin-protein ligase HACE1
E3 ubiquitin-protein ligase RING1
EBF transcription factor 1;EBF transcription factor 3;EBF transcription factor 3;EBF transcription factor 1;EBF transcription factor 3;EBF transcription factor 2
Ecm29 proteasome adaptor and scaffold
Ecotropic viral integration site 5 like
Ectonucleotide pyrophosphatase/phosphodiesterase 7
Ectonucleotide pyrophosphatase/phosphodiesterase family member 5
EF-hand and coiled-coil domain containing 1
EF-hand domain family member D1
EGF like domain multiple 7
ELMO domain containing 1
ELMO domain containing 2
Elongation factor G, mitochondrial;Elongation factor G, mitochondrial;Elongation factor G, mitochondrial;Elongation factor G, mitochondrial
Elongation factor like GTPase 1
Elongation factor Ts, mitochondrial
Emerin
EMI domain containing 1
Endoplasmic reticulum lectin 1
Endoplasmic reticulum protein SC65
Endothelial cell adhesion molecule
Enhancer of mRNA-decapping protein 4
Enoyl-CoA hydratase
Enoyl-CoA hydratase domain containing 2
Enoyl-CoA hydratase domain containing 3
Ephrin B1;Ephrin-B1;Ephrin-B1
Epidermal growth factor receptor pathway substrate 15 like 1
Epsilon-sarcoglycan
Epsin 2
ER membrane protein complex subunit 7
ER membrane protein complex subunit 8;MPN domain-containing protein

ERO1-like protein alpha
Erythrocyte membrane protein band 4.1 like 3
Eukaryotic initiation factor 4A-III
Eukaryotic translation initiation factor 1
Eukaryotic translation initiation factor 2 alpha kinase 2
Eukaryotic translation initiation factor 2A
Eukaryotic translation initiation factor 2B subunit delta
Eukaryotic translation initiation factor 4B isoform 1
Eukaryotic translation initiation factor 4C
EWS RNA binding protein 1
Exo/endonuclease G
Exocyst complex component 5
Exocyst complex component 7
Exocyst complex component 8
Exosome complex component RRP41
Exosome component 5
Exosome RNA helicase MTR4
Extended synaptotagmin 2
F-actin monooxygenase
F-box and leucine rich repeat protein 20
FAD-binding FR-type domain-containing protein
FAD-binding PCMH-type domain-containing protein
Family with sequence similarity 111 member A
Family with sequence similarity 91 member A1
Family with sequence similarity 98 member B
Fasciculation and elongation protein zeta 1
Fatty acid desaturase 2
FERM, ARH/RhoGEF and pleckstrin domain protein 1;FERM, ARH/RhoGEF and pleckstrin domain protein 1;FERM, ARH/RhoGEF and pleckstrin domain protein 1
Fetuin B
FGE-sulfatase domain-containing protein;Sulfatase-modifying factor 2 isoform b
Fibronectin type III domain containing 1
Fibronectin type III domain containing 3B
Fibulin 7
Filamin B
Filamin binding LIM protein 1
Follistatin-like protein 1
Formin like 3
Formyltetrahydrofolate synthetase
Fructose-bisphosphatase
Fucose kinase
G protein beta subunit-like

G protein signaling modulator 1;G-protein-signaling modulator 1;G protein signaling modulator 1
G protein signaling modulator 2
G3BP stress granule assembly factor 1
G3BP stress granule assembly factor 2
Galactosylgalactosylxylosylprotein 3-beta-glucuronosyltransferase
Gamma-interferon-inducible protein 16 isoform 1;Uncharacterized protein;Uncharacterized protein;Uncharacterized protein
Gamma-sarcoglycan
Gamma-tubulin complex component
Ganglioside induced differentiation associated protein 1;Ganglioside-induced differentiation-associated protein 1
GATOR complex protein MIOS isoform X1;Meiosis regulator for oocyte development;GATOR complex protein MIOS isoform X1;Meiosis regulator for oocyte development
GDNF family receptor alpha-1;GDNF family receptor alpha 1;GDNF family receptor alpha-1
GDNF family receptor alpha-2
GDP-D-mannose dehydratase
GDP-fucose protein O-fucosyltransferase 1
GDP-Man:Man(3)GlcNAc(2)-PP-Dol alpha-1,2-mannosyltransferase
General transcription factor IIi;General transcription factor IIi;General transcription factor IIi;General transcription factor II-I isoform 1;General transcription factor IIi
GH3 domain containing
Gliomedin
Glucose transporter type 4, insulin-responsive;Glucose transporter type 4, insulin-responsive;Glucose transporter type 4, insulin-responsive
Glutamate receptor
Glutaryl-CoA dehydrogenase isoform a
Glutathione peroxidase
Glutathione peroxidase
Glutathione S-transferase kappa
Glycerol-3-phosphate acyltransferase 4
Glycine C-acetyltransferase
Glycosyltransferase 25 domain containing 1
Glycosyltransferase 8 domain containing 2
Glyoxylate reductase 1 homolog
Golgi brefeldin A resistant guanine nucleotide exchange factor 1
Golgi SNAP receptor complex member 1;28 kDa Golgi SNARE protein;Golgi SNAP receptor complex member 1;28 kDa Golgi SNARE protein;Golgi SNAP receptor complex member 1
Golgin B1
GRAM domain containing 1B
GRB2 associated binding protein 1
Gremlin 1, DAN family BMP antagonist;Gremlin
GrpE protein homolog
GTP-binding protein 1
GTPase IMAP family member 4

Guanine nucleotide exchange factor H1;Guanine nucleotide exchange factor H1;Rho guanine nucleotide exchange factor 2;Guanine nucleotide exchange factor H1;Guanine nucleotide exchange factor H1;Guanine nucleotide exchange factor H1;Guanine nucleotide exchange factor H1
Guided entry of tail-anchored proteins factor 4
Haloacid dehalogenase-like hydrolase domain-containing protein 3
HEAT repeat-containing protein 1
Heat shock protein beta-8
HECT domain E3 ubiquitin protein ligase 3
HECT domain E3 ubiquitin protein ligase 4
HECT domain-containing protein;HECT domain-containing protein;HECT domain-containing protein;HECT domain-containing protein;HECT domain-containing protein;HECT domain-containing protein;HECT domain-containing protein;Uncharacterized protein;HECT domain-containing protein
HECT-type E3 ubiquitin transferase
Heterogeneous nuclear ribonucleoprotein H2
Heterogeneous nuclear ribonucleoprotein L like
Heterogeneous nuclear ribonucleoprotein U like 1
Hexose-6-phosphate dehydrogenase/glucose 1-dehydrogenase;GDH/6PGL endoplasmic bifunctional protein isoform 1;Hexose-6-phosphate dehydrogenase/glucose 1-dehydrogenase
High mobility group nucleosome-binding domain-containing protein 3;Non-histone chromosomal protein HMG-17;High mobility group nucleosome-binding domain-containing protein 3;High mobility group nucleosome-binding domain-containing protein 3
High mobility group protein B2
Histone deacetylase 1
Histone deacetylase 2
Histone deacetylase 6
Histone domain-containing protein
Hook microtubule tethering protein 3
Host cell factor C2
Huntingtin interacting protein 1 related
Hydroxyacyl-coenzyme A dehydrogenase, mitochondrial
Hydroxymethylglutaryl-CoA lyase
Hydroxysteroid 17-beta dehydrogenase 3
Ig-like domain-containing protein
Ig-like domain-containing protein;Uncharacterized protein;Ig-like domain-containing protein
Immunity related GTPase Q
Importin subunit alpha
Indolethylamine N-methyltransferase isoform 1
Inner nuclear membrane protein Man1 isoform 1;LEM domain containing 3;LEM domain containing 3;LEM domain containing 3
Inorganic diphosphatase
Inositol polyphosphate 1-phosphatase isoform X1
Insulin like growth factor binding protein 7
Insulin-like growth factor-binding protein 6

Integral membrane protein 2
Integral membrane protein 2
Integrator complex subunit 5
Integrin beta
Integrin subunit alpha 7
Integrin subunit alpha L
Integrin subunit beta like 1
Intercellular adhesion molecule 1
Interferon-induced GTP-binding protein Mx1
Interferon-induced GTP-binding protein Mx2
Interferon-related developmental regulator 2
Iron-sulfur cluster assembly enzyme
Iron-sulfur cluster assembly factor IBA57
Isochorismatase domain containing 2
ITPR interacting domain containing 2
Junctional adhesion molecule 2
KH RNA binding domain containing, signal transduction associated 1
KH RNA binding domain containing, signal transduction associated 3;KH RNA binding domain containing, signal transduction associated 3
KIAA1217
Kinase suppressor of ras 1
Kinesin light chain
Kinesin light chain;Kinesin light chain 2
Kinesin-like protein
KN motif and ankyrin repeat domains 2
L-serine ammonia-lyase
La ribonucleoprotein 1, translational regulator;La ribonucleoprotein 1, translational regulator;La ribonucleoprotein 1, translational regulator
Lambda-crystallin homolog
Late endosomal/lysosomal adaptor, MAPK and MTOR activator 2
Latent transforming growth factor beta binding protein 4
Latent-transforming growth factor beta-binding protein 2;Latent transforming growth factor beta binding protein 2;Latent transforming growth factor beta binding protein 2;Latent transforming growth factor beta binding protein 2;Latent transforming growth factor beta binding protein 2;Latent transforming growth factor beta binding protein 2
LDL receptor related protein 5;Low-density lipoprotein receptor-related protein;Low-density lipoprotein receptor-related protein;Low-density lipoprotein receptor-related protein
LDLR chaperone MESD
Leiomodin-1;Leiomodin 1
Leucine rich repeat containing 59
Leucine rich repeat containing 8 VRAC subunit A
Leucine rich repeat LGI family member 4
Leucine rich repeat transmembrane neuronal 1

Leucine-rich repeat protein SHOC-2
Leucine-rich repeat-containing protein 14
Leukocyte cell-derived chemotaxin-2;Leukocyte cell derived chemotaxin 2
LIM and cysteine-rich domains protein 1
LIM and senescent cell antigen-like-containing domain protein
LIM domain 7
LIM domain-containing protein 1
Lipocalin 2
Lipoprotein lipase
Lon protease homolog 2, peroxisomal;Lon protease homolog 2, peroxisomal;Lon protease homolog 2, peroxisomal
LRR binding FLII interacting protein 2
LRRcap domain-containing protein
LRRCT domain-containing protein
LUC7 like 3 pre-mRNA splicing factor
Lys-63-specific deubiquitinase
Lysocardiolipin acyltransferase 1;Lysocardiolipin acyltransferase 1;Lysocardiolipin acyltransferase 1;1-acylglycerol-3-phosphate O-acyltransferase 8
Lysophosphatidylcholine acyltransferase 1
Lysophosphatidylcholine acyltransferase 2
Lysozyme
Lysyl oxidase homolog
Maestro heat like repeat family member 1
MAP7 domain containing 1
MAP7 domain containing 2
Matrix AAA peptidase interacting protein 1
Matrix Gla protein
Mediator of RNA polymerase II transcription subunit 1
Melanoma inhibitory activity protein 3 isoform X6;Transport and Golgi organization protein 1 homolog isoform 1;SH3 domain-containing protein
Membrane magnesium transporter
Metalloproteinase inhibitor 2
Metallothionein
Metastasis associated 1
Metastasis associated 1 family member 2
Metaxin
Metaxin-1
Metaxin-2
Methionine--tRNA ligase, cytoplasmic;Methionine--tRNA ligase, cytoplasmic;Methionine--tRNA ligase, cytoplasmic;Methionine--tRNA ligase, cytoplasmic
Methyl-CpG-binding protein 2;Methyl-CpG-binding protein 2;Methyl-CpG binding protein 2
Methylcrotonoyl-CoA carboxylase 2
Microsomal prostaglandin E synthase 2

Microsomal signal peptidase 12 kDa subunit
Microtubule associated protein 6
Microtubule-associated protein
Mitochondrial amidoxime-reducing component 1;MOSC domain-containing protein
Mitochondrial calcium uptake 1;Mitochondrial calcium uptake 1;Calcium uptake protein 1, mitochondrial isoform 2;Mitochondrial calcium uptake 1
Mitochondrial fission factor
Mitochondrial fission regulator 1 like
Mitochondrial glutamate carrier 1 isoform X1
Mitochondrial import inner membrane translocase subunit Tim21
Mitochondrial import inner membrane translocase subunit TIM44;Translocase of inner mitochondrial membrane 44;Translocase of inner mitochondrial membrane 44
Mitochondrial import inner membrane translocase subunit TIM50
Mitochondrial ribosomal protein L10
Mitochondrial ribosomal protein L19;Mitochondrial ribosomal protein L19;39S ribosomal protein L19, mitochondrial
Mitochondrial ribosomal protein L39;Mitochondrial ribosomal protein L39;39S ribosomal protein L39, mitochondrial isoform a;Mitochondrial ribosomal protein L39
Mitochondrial ribosomal protein L4
Mitochondrial ribosomal protein L41
Mitochondrial ribosomal protein L47
Mitochondrial ribosomal protein L58
Mitochondrial ribosomal protein S34
Mitogen-activated protein kinase 2-associated protein 1;Mitogen-activated protein kinase 2-associated protein 1;Mitogen-activated protein kinase 2-associated protein 1;MAPK associated protein 1
Mitogen-activated protein kinase kinase kinase kinase 4
Mitogen-activated protein kinase;Mitogen-activated protein kinase 14;Mitogen-activated protein kinase;Mitogen-activated protein kinase 14;Mitogen-activated protein kinase;Mitogen-activated protein kinase
Mitogen-activated protein kinase;Mitogen-activated protein kinase;Mitogen-activated protein kinase;Mitogen-activated protein kinase;Mitogen-activated protein kinase 9;Mitogen-activated protein kinase;Mitogen-activated protein kinase;Mitogen-activated protein kinase;Mitogen-activated protein kinase;Mitogen-activated protein kinase;Mitogen-activated protein kinase;Mitogen-activated protein kinase;Mitogen-activated protein kinase;Mitogen-activated protein kinase;Mitogen-activated protein kinase;Mitogen-activated protein kinase;Mitogen-activated protein kinase;Mitogen-activated protein kinase;Mitogen-activated protein kinase;Mitogen-activated protein kinase
Modulator of non-genomic activity of estrogen receptor
Mono-ADP ribosylhydrolase 1
Monocyte differentiation antigen CD14
MRP-S28 domain-containing protein;28S ribosomal protein S35, mitochondrial isoform 1
Muellerian-inhibiting factor
Multimerin 2

Muscleblind 2-like protein;Muscleblind like splicing regulator 1;Muscleblind like splicing regulator 2;Muscleblind like splicing regulator 1;Muscleblind like splicing regulator 1;Muscleblind like splicing regulator 1;Muscleblind like splicing regulator 1;Muscleblind like splicing regulator 1;Muscleblind like splicing regulator 2;Muscleblind like splicing regulator 1;Muscleblind like splicing regulator 2;Muscleblind like splicing regulator 3;Muscleblind like splicing regulator 1;Muscleblind like splicing regulator 2;Muscleblind like splicing regulator 2;Muscleblind like splicing regulator 2;Muscleblind like splicing regulator 2;Muscleblind like splicing regulator 2;Muscleblind like splicing regulator 1;Muscleblind like splicing regulator 3;Muscleblind like splicing regulator 3;Muscleblind like splicing regulator 3;Muscleblind like splicing regulator 3
MYB binding protein 1a
Myeloid cell nuclear differentiation antigen
Myosin IF
Myosin IXB
Myosin phosphatase Rho interacting protein
N-acetyl-alpha-glucosaminidase
N-acetylated alpha-linked acidic dipeptidase 2
N-acetylglucosamine-6-sulfatase
N-alpha-acetyltransferase 15, NatA auxiliary subunit;N-alpha-acetyltransferase 15, NatA auxiliary subunit;N-alpha-acetyltransferase 15, NatA auxiliary subunit
N-glycanase 1
NACHT domain-containing protein;NLR family member X1 isoform X1
NADH dehydrogenase [ubiquinone] 1 alpha subcomplex assembly factor 3
NADH dehydrogenase [ubiquinone] 1 alpha subcomplex assembly factor 4
NADH dehydrogenase [ubiquinone] 1 beta subcomplex subunit 8, mitochondrial
NADH dehydrogenase [ubiquinone] iron-sulfur protein 4, mitochondrial
Nebulette
NEDD8
Negative regulator of ubiquitin like proteins 1
Nerve growth factor receptor
Neural Wiskott-Aldrich syndrome protein;WASP like actin nucleation promoting factor;WASP like actin nucleation promoting factor
Neuropilin
Neuroplastin
NF-kappa-B essential modulator
Nicotinamide-nucleotide adenylyltransferase
Noelin isoform 4
Non-specific serine/threonine protein kinase
Non-specific serine/threonine protein kinase;Non-specific serine/threonine protein kinase;Non-specific serine/threonine protein kinase;Serine/threonine kinase 3;Serine/threonine kinase 4;Non-specific serine/threonine protein kinase;Non-specific serine/threonine protein kinase;Non-specific serine/threonine protein kinase;Non-specific serine/threonine protein kinase
Nuclear cap binding protein subunit 1
Nuclear cap binding protein subunit 1
Nuclear cap-binding protein subunit 2
Nuclear casein kinase and cyclin dependent kinase substrate 1
Nuclear distribution protein nudE-like 1

Nuclear export mediator factor
Nuclear factor 1
Nuclear factor NF-kappa-B p100 subunit isoform X1;Nuclear factor kappa B subunit 2
Nuclear mitotic apparatus protein 1
Nuclear pore complex protein
Nuclear pore complex protein Nup85
Nuclear pore complex protein Nup96
Nuclear receptor binding protein 1
Nuclear receptor coactivator 5
Nuclear receptor coactivator 7
Nuclear respiratory factor 1
Nucleoporin 133
Nucleoporin 153;Nuclear pore complex protein Nup153 isoform 2;Nucleoporin 153
Nucleoporin 160
Nucleoporin 205
Nucleoporin 43
Nucleoporin 54
Nucleoporin 62
Nucleoporin 88
Nucleoporin NUP53
Nucleoside-diphosphate kinase
Nucleotide binding protein like
O-GlcNAc transferase subunit p110
Occludin
Opioid growth factor receptor
Optineurin
ORM1-like protein;ORM1-like protein 3;ORM1-like protein
OS9 endoplasmic reticulum lectin
Out at first protein homolog
OXA1L mitochondrial inner membrane protein
Oxysterol-binding protein
Palmdelphin
Paraoxonase
Paraspeckle component 1
Patatin like phospholipase domain containing 6
Patatin like phospholipase domain containing 7
Patatin like phospholipase domain containing 8;Calcium-independent phospholipase A2-gamma isoform 1
PCI domain containing 2
PDS5 cohesin associated factor B
PDZ and LIM domain 4
PDZ and LIM domain 7
PDZ domain-containing protein 11

Pecanex-like protein
Pentatricopeptide repeat domain 3
Pentraxin-3
Peptidase inhibitor 16
Peptide-O-fucosyltransferase
Peptidyl-prolyl cis-trans isomerase
Peptidyl-prolyl cis-trans isomerase
Peptidylprolyl isomerase
Perilipin 5
Periostin
Peroxin-14
Peroxisomal trans-2-enoyl-CoA reductase
PH domain-containing protein
PH domain-containing protein;Pleckstrin homology domain-containing family O member 2 isoform 1;PH domain-containing protein;PH domain-containing protein;PH domain-containing protein;PH domain-containing protein;Uncharacterized protein
PHD finger protein 2
Phosphatidylinositol 4-kinase alpha
Phosphatidylinositol-3,4-bisphosphate 4-phosphatase;Phosphatidylinositol-3,4-bisphosphate 4-phosphatase;Phosphatidylinositol-3,4-bisphosphate 4-phosphatase;Phosphatidylinositol-3,4-bisphosphate 4-phosphatase
Phosphatidylinositol-3,4,5-trisphosphate 3-phosphatase
Phosphatidylinositol-4-phosphate 3-kinase
Phosphatidylserine decarboxylase proenzyme, mitochondrial;Phosphatidylserine decarboxylase proenzyme, mitochondrial;Phosphatidylserine decarboxylase;Phosphatidylserine decarboxylase proenzyme, mitochondrial;Phosphatidylserine decarboxylase;Phosphatidylserine decarboxylase proenzyme, mitochondrial
Phosphoglucomutase 5
Phospholipase A2 activating protein
Phospholipase B-like
Phospholipase D family member 4
Phosphorylase b kinase regulatory subunit
Phosphorylase b kinase regulatory subunit
Phytanoyl-CoA 2-hydroxylase
Plakophilin 4
Plasma retinol-binding protein;Retinol-binding protein
Plasma serine protease inhibitor preproprotein;Serpin family A member 5
Plasmalemma vesicle associated protein
Platelet-derived growth factor receptor-like protein
Pleckstrin homology and FYVE domain containing 1
Pleckstrin homology domain containing A5
Pleckstrin homology domain interacting protein
Plectin
Plectin

Pleiotrophin
Plexin A4
PML nuclear body scaffold
Polyhomeotic homolog 2
Polypeptide N-acetylgalactosaminyltransferase
Polypeptide N-acetylgalactosaminyltransferase;Ricin B-type lectin domain-containing protein
Polyprenol reductase
PPFIA binding protein 1
Pre-B-cell leukemia transcription factor-interacting protein 1 isoform 1;Uncharacterized protein
Pre-mRNA-processing factor 31
Prefoldin subunit 2
Prelamin-A/C
Prenylcysteine oxidase 1 like
Prickle planar cell polarity protein 1
Pro-apoptotic WT1 regulator
Procollagen-lysine 5-dioxygenase
Procollagen-proline 3-dioxygenase
Procollagen-proline 3-dioxygenase;Prolyl 3-hydroxylase 2;Procollagen-proline 3-dioxygenase
Programmed cell death protein 4
Prolyl-tRNA synthetase
Prolylcarboxypeptidase
Propionate--CoA ligase
Prostaglandin reductase 3 isoform 1
Proteasome subunit beta
Proteasome subunit beta;Proteasome endopeptidase complex;Proteasome subunit beta;Proteasome endopeptidase complex
Protein activator of interferon induced protein kinase EIF2AK2
Protein BUD31 homolog
Protein C receptor
Protein canopy homolog 3
Protein disulfide isomerase family A member 5
Protein disulfide-isomerase
Protein FAM3C
Protein HGH1 homolog
Protein kinase AMP-activated non-catalytic subunit beta 2;5'-AMP-activated protein kinase subunit beta-2;Protein kinase AMP-activated non-catalytic subunit beta 2
Protein O-glucosyltransferase 1
Protein O-glucosyltransferase 2
Protein phosphatase 1 regulatory subunit
Protein phosphatase 1 regulatory subunit 9B
Protein phosphatase, Mg2+/Mn2+ dependent 1L
Protein quaking

Protein RFT1 homolog
Protein S100
Protein transport protein SEC23
Protein tyrosine phosphatase 4A2
Protein VAC14 homolog
Protein-ribulosamine 3-kinase
Protein-serine/threonine kinase
Protein-tyrosine sulfotransferase
Protoporphyrinogen oxidase
PRP6 homolog
Pseudopodium enriched atypical kinase 1
PTPRF interacting protein alpha 1
PWWP domain-containing protein
Pyridoxal 5'-phosphate synthase
Pyrroline-5-carboxylate reductase
Pyruvate dehyrogenase phosphatase catalytic subunit 1
RAB, member of RAS oncogene family like 3;RAB, member of RAS oncogene family like 3;RAB, member of RAS oncogene family like 3;RAB, member of RAS oncogene family like 3
RAB3 GTPase activating non-catalytic protein subunit 2
RAB30, member RAS oncogene family
RAB6B, member RAS oncogene family
Rabenosyn, RAB effector
RAD50 interactor 1
Rae1 protein homolog
Ral GTPase-activating protein subunit alpha-1
RAN binding protein 1
RAN binding protein 3
Ras interacting protein 1
Ras like without CAAX 1
RAS p21 protein activator 3
Ras-related GTP-binding protein
RB binding protein 7, chromatin remodeling factor;RB binding protein 7, chromatin remodeling factor;RB binding protein 7, chromatin remodeling factor;RB binding protein 7, chromatin remodeling factor;RB binding protein 7, chromatin remodeling factor
Receptor expression-enhancing protein
Receptor expression-enhancing protein
Receptor expression-enhancing protein
Receptor interacting serine/threonine kinase 1
Receptor protein-tyrosine kinase
Receptor tyrosine kinase like orphan receptor 2
Reelin
Regulation of nuclear pre-mRNA domain containing 1B

Regulator of cell cycle
Regulator of microtubule dynamics 1
Regulator of microtubule dynamics 2
Regulator of microtubule dynamics 3
Replication protein A subunit
Replication protein A3
Replication stress response regulator SDE2
Required for meiotic nuclear division 1 homolog
Ret proto-oncogene;Proto-oncogene tyrosine-protein kinase receptor Ret
Reticulocalbin 2
Reticulocalbin 3
Reticulon
Reticulon 4 interacting protein 1
Reticulophagy regulator 1
Reticulophagy regulator 3
Retinol dehydrogenase 13
Retinol dehydrogenase 5
RGS domain-containing protein;RGS domain-containing protein;Uncharacterized protein;Uncharacterized protein;RGS domain-containing protein
Rho GDP dissociation inhibitor beta
Rho GTPase activating protein 31;Rho GTPase activating protein 31;Rho GTPase-activating protein 31
Rho GTPase-activating protein 35
Rho guanine nucleotide exchange factor 10 like
Rho guanine nucleotide exchange factor 12
Rho guanine nucleotide exchange factor 17
Rho guanine nucleotide exchange factor 7
Rho related BTB domain containing 3
Rho-GAP domain-containing protein
Ribonucleoside-diphosphate reductase;Ribonucleoside-diphosphate reductase;Ribonucleoside-diphosphate reductase;Ribonucleotide reductase catalytic subunit M1
Ribonucloprotein
Ribosomal protein S6 kinase
Ribosomal protein S6 kinase;Ribosomal protein S6 kinase;Non-specific serine/threonine protein kinase;Ribosomal protein S6 kinase;Non-specific serine/threonine protein kinase;Non-specific serine/threonine protein kinase;Ribosomal protein S6 kinase;Non-specific serine/threonine protein kinase;Non-specific serine/threonine protein kinase;Non-specific serine/threonine protein kinase
Ribosomal RNA processing 12 homolog
Ribosomal_L2_C domain-containing protein;Ribosomal_L2 domain-containing protein
Ribosome production factor 2 homolog
Ring finger protein 7
RNA binding motif protein 10
RNA binding motif protein 14

RNA binding motif protein 26;RNA binding motif protein 26;RNA binding motif protein 26;RNA binding motif protein 26;RNA-binding protein 27 isoform X7;RNA binding motif protein 26;RNA binding motif protein 26
RNA binding motif protein 39
RNA binding motif single stranded interacting protein 3;RNA binding motif single stranded interacting protein 3;RNA binding motif single stranded interacting protein 1;RNA binding motif single stranded interacting protein 1;RNA binding motif single stranded interacting protein 3;RNA binding motif single stranded interacting protein 1;RNA binding motif single stranded interacting protein 1;RNA binding motif single stranded interacting protein 3;RNA binding motif single stranded interacting protein 1;RNA binding motif single stranded interacting protein 3
RNA helicase
RNA-binding protein 4B;RNA binding motif protein 14
RNA-binding protein 8A
RNA-binding protein with serine-rich domain 1
RU2B
RUN and FYVE domain containing 1
RUN and FYVE domain containing 2
RUN domain containing 3A
S1 RNA binding domain 1
Sacsin isoform X1;Uncharacterized protein;Uncharacterized protein
SAP domain-containing protein;Protein DEK
Saposin B-type domain-containing protein
Scaffold attachment factor B
Scavenger receptor cysteine rich family member with 5 domains
Scavenger receptor cysteine-rich type 1 protein M130
Sec1 family domain containing 1
Sec1 family domain containing 2
SEC23 interacting protein
SECIS binding protein 2 like
Secreted frizzled-related protein 4
Secretory carrier-associated membrane protein
Selenoprotein I
Sema domain-containing protein;Uncharacterized protein;Uncharacterized protein;Sema domain-containing protein
Semaphorin 3B
Septin
Serine and arginine repetitive matrix 1
Serine and arginine rich splicing factor 12;Serine and arginine rich splicing factor 10
Serine/arginine repetitive matrix 2
Serine/threonine kinase 24
Serine/threonine-protein kinase
Serine/threonine-protein kinase mTOR
Serine/threonine-protein phosphatase
Serine/threonine-protein phosphatase 2A 56 kDa regulatory subunit

Serine/threonine-protein phosphatase 2A 56 kDa regulatory subunit;Serine/threonine-protein phosphatase 2A 56 kDa regulatory subunit;Protein phosphatase 2 regulatory subunit B'gamma;Protein phosphatase 2 regulatory subunit B'gamma;Serine/threonine-protein phosphatase 2A 56 kDa regulatory subunit
Serotransferrin
SH3 domain containing GRB2 like 2, endophilin A1;SH3 domain containing GRB2 like 2, endophilin A1
Short-chain-specific acyl-CoA dehydrogenase, mitochondrial
Sideroflexin-1;Sidoreflexin;Sidoreflexin
Signal peptide, CUB domain and EGF like domain containing 1
Signal recognition particle 19
Signal sequence receptor subunit gamma
SLAIN motif family member 2
Slit guidance ligand 3
Small nuclear ribonucleoprotein U5 subunit 40
Smoothelin
SMU1 DNA replication regulator and spliceosomal factor
Sodium/potassium-transporting ATPase subunit alpha
Solute carrier family 25 member 17
Solute carrier family 25 member 51
Solute carrier family 30 member 1
Solute carrier family 30 member 9
Solute carrier family 35 member F6
Sorbin and SH3 domain-containing protein 2
Sortilin 1
Sortilin related VPS10 domain containing receptor 1
Sorting nexin 13
Sorting nexin 14
Sorting nexin 18;Sorting nexin 18;Sorting nexin 18;Sorting nexin 18;Sorting nexin
Sorting nexin 24
Sorting nexin 33;Sorting nexin
Sorting nexin-17
SPARC like 1;SPARC like 1;SPARC like 1;SPARC-like protein 1;SPARC-like protein 1
SPARC related modular calcium binding 1
Spartin
Spectrin alpha, non-erythrocytic 1
Spermatogenesis associated serine rich 2 like;Spermatogenesis associated serine rich 2 like;Spermatogenesis associated serine rich 2 like;Spermatogenesis associated serine rich 2 like;Spermatogenesis associated serine rich 2 like;SPATS2-like protein isoform d;Spermatogenesis associated serine rich 2 like
SPG7 matrix AAA peptidase subunit, paraplegin;SPG7 matrix AAA peptidase subunit, paraplegin;Paraplegin
Sphingomyelin phosphodiesterase acid like 3B
Sphingosine kinase 2
Sphingosine-1-phosphate phosphatase 1
Spire type actin nucleation factor 1

Spliceosome associated factor 3, U4/U6 recycling protein;Spliceosome associated factor 3, U4/U6 recycling protein;Spliceosome associated factor 3, U4/U6 recycling protein;Spliceosome associated factor 3, U4/U6 recycling protein;Spliceosome associated factor 3, U4/U6 recycling protein
Spliceosome-associated protein CWC15 homolog
Splicing factor 3a subunit 3;Splicing factor 3A subunit 3 isoform 1
Splicing factor 3b subunit 2;Splicing factor 3b subunit 2;Splicing factor 3B subunit 2
Splicing factor 3b subunit 4
Splicing factor 3B subunit 6
Splicing regulatory glutamic acid and lysine rich protein 1
SpoU_methylase domain-containing protein
SR-related CTD associated factor 8;SR-related CTD associated factor 8;SR-related CTD associated factor 8;SR-related CTD associated factor 4;SR-related CTD associated factor 4;SR-related CTD associated factor 4;SR-related CTD associated factor 8;SR-related CTD associated factor 4
SRA stem-loop interacting RNA binding protein
SRSF protein kinase 2
StAR related lipid transfer domain containing 13
Stathmin
Staufen double-stranded RNA binding protein 1;Staufen double-stranded RNA binding protein 1;Staufen double-stranded RNA binding protein 1;Staufen double-stranded RNA binding protein 1;Double-stranded RNA-binding protein Staufen homolog 1 isoform b;Staufen double-stranded RNA binding protein 1;Staufen double-stranded RNA binding protein 1
Sterol 26-hydroxylase, mitochondrial;Uncharacterized protein
Stimulator of chondrogenesis 1
Stonin-2
Store-operated calcium entry-associated regulatory factor
Striatin
Stromal antigen 1;Stromal antigen 1;Cohesin subunit SA-1;Stromal antigen 1;Stromal antigen 1
Stromal cell derived factor 2;Stromal cell-derived factor 2
Structural maintenance of chromosomes flexible hinge domain containing 1
Succinate dehydrogenase [ubiquinone] cytochrome b small subunit
Sulfate transporter
Sulfhydryl oxidase
Sulfotransferase
SUMO peptidase family member, NEDD8 specific
Supervillin
SURF1-like protein
SWI/SNF related, matrix associated, actin dependent regulator of chromatin subfamily c member 2;SWI/SNF related, matrix associated, actin dependent regulator of chromatin subfamily c member 2;SWI/SNF related, matrix associated, actin dependent regulator of chromatin subfamily c member 2
SWI/SNF related, matrix associated, actin dependent regulator of chromatin, subfamily e, member 1;SWI/SNF related, matrix associated, actin dependent regulator of chromatin, subfamily e, member 1;SWI/SNF related, matrix associated, actin dependent regulator of chromatin, subfamily e, member 1;SWI/SNF related, matrix associated, actin dependent regulator of chromatin, subfamily e, member 1;SWI/SNF related, matrix associated, actin dependent regulator of chromatin, subfamily e, member 1
SWIB domain-containing protein

Synapse defective Rho GTPase homolog 1
Synaptic functional regulator FMR1
Synaptopodin
Synaptotagmin
Synaptotagmin 11;Synaptotagmin-11
Syndecan
Synemin
Syntaxin 6
Synthesis of cytochrome C oxidase 1
T-complex 11 like 1
Target of EGR1, exonuclease
TATA-box binding protein associated factor 9;TATA-box binding protein associated factor 9b;TATA-box binding protein associated factor 9b;TATA-box binding protein associated factor 9b
TBC1 domain family member 23
Telomeric repeat-binding factor 2
Telomeric repeat-binding factor 2-interacting protein 1
Tensin 2
Testis expressed 10
Testis expressed 2
Testis expressed 264, ER-phagy receptor
Tetraspanin
Tetratricopeptide repeat domain 7B
Tetratricopeptide repeat domain 9
Tetratricopeptide repeat protein 37 isoform X1
THAP domain containing 11
Thioredoxin domain containing 9
Thioredoxin domain-containing protein 12
THO complex 6
THO complex subunit 2
Thymocyte nuclear protein 1
Tight junction protein 1
TIP120 domain-containing protein
TLC domain-containing protein;TLC domain-containing protein;TLC domain-containing protein;Uncharacterized protein
TNF alpha induced protein 6
TNF receptor associated factor 2;TNF receptor-associated factor
TNF receptor-associated factor
Torsin 1A interacting protein 2
Trafficking protein particle complex 11;Trafficking protein particle complex subunit 11
Trafficking protein particle complex 6B
Transcription elongation factor SPT4
Transcription elongation regulator 1

Transcription factor A, mitochondrial
Transducin beta like 2
Transferrin receptor protein 1
Transformation/transcription domain associated protein
Transforming growth factor beta receptor type 3
Transglutaminase 2
Transient receptor potential cation channel subfamily M member 4
Transmembrane p24 trafficking protein 1
Transmembrane protein 11
Transmembrane protein 120A isoform 1
Transmembrane protein 126A isoform 1
Transmembrane protein 177
Transmembrane protein 214
Transmembrane protein 87A
Transmembrane protein with metallophosphoesterase domain
Transportin 2
Treacle ribosome biogenesis factor 1
Tripartite motif containing 2
Tripartite motif containing 3
Tripartite motif containing 47
Tropomyosin 2
TSC complex subunit 2
Tubulin gamma chain
Tumor protein D52
Twinfilin actin binding protein 2
Tyrosine-protein kinase
Tyrosine-protein kinase receptor
Tyrosine-protein kinase receptor;Neurotrophic receptor tyrosine kinase 2;Tyrosine-protein kinase receptor;BDNF/NT-3 growth factors receptor isoform X5;Tyrosine-protein kinase receptor
Tyrosine-protein phosphatase non-receptor type;Tyrosine-protein phosphatase non-receptor type;Protein-tyrosine-phosphatase
U2 snRNP associated SURP domain containing
U6 snRNA-associated Sm-like protein LSm4
Ubiquilin 2
Ubiquinone biosynthesis monooxygenase COQ6, mitochondrial;Ubiquinone biosynthesis monooxygenase COQ6, mitochondrial;Ubiquinone biosynthesis monooxygenase COQ6, mitochondrial
Ubiquinone biosynthesis O-methyltransferase, mitochondrial;Ubiquinone biosynthesis O-methyltransferase, mitochondrial
Ubiquinone biosynthesis protein
Ubiquitin associated and SH3 domain containing B
Ubiquitin carboxyl-terminal hydrolase 46 isoform 1;Ubiquitin specific peptidase 46;Ubiquitin specific peptidase 46
Ubiquitin specific peptidase 39;Ubiquitin specific peptidase 39;U4/U6.U5 tri-snRNP-associated protein 2;Ubiquitin specific peptidase 39

Ubiquitin-fold modifier-conjugating enzyme 1
Ubiquitinyl hydrolase 1
UBX domain protein 4
UDP-glucose glycoprotein glucosyltransferase 2
UDP-glucuronosyltransferase
UDP-N-acetylglucosamine pyrophosphorylase 1
UDP-N-acetylglucosamine pyrophosphorylase 1 like 1
Ufm1-specific protease 2
Uncharacterized protein
Uncharacterized protein;Agenet-like domain-containing protein;Agenet-like domain-containing protein;Agenet-like domain-containing protein;Agenet-like domain-containing protein;Agenet-like domain-containing protein;Uncharacterized protein;Fragile X mental retardation syndrome-related protein 1 isoform X1
Uncharacterized protein;Arf-GAP with GTPase, ANK repeat and PH domain-containing protein 1 isoform X7;Uncharacterized protein
Uncharacterized protein;Complement component C1q receptor
Uncharacterized protein;Golgi phosphoprotein 3
Uncharacterized protein;Neurogenic locus notch homolog protein 2 isoform 1 preproprotein;Uncharacterized protein
Uncharacterized protein;Prefoldin subunit 3;Uncharacterized protein;Prefoldin subunit 3;Uncharacterized protein
Uncharacterized protein;TSC22 domain family protein 4 isoform a
Uncharacterized protein;Uncharacterized protein;Uncharacterized protein;Nucleolar transcription factor 1 isoform X1
Uncharacterized protein;Uncharacterized protein;Uncharacterized protein;Uncharacterized protein;Complement C3
USP1-associated factor 1
UTP--glucose-1-phosphate uridylyltransferase
Vacuolar protein sorting 33A homolog
Vacuolar protein sorting-associated protein 28 homolog
Vacuolar protein sorting-associated protein 41 homolog
Vacuolar protein sorting-associated protein 52 homolog
Vacuolar protein sorting-associated protein 53 homolog
Vascular cell adhesion molecule 1
Vasodilator stimulated phosphoprotein;Vasodilator stimulated phosphoprotein;Vasodilator-stimulated phosphoprotein
Very low-density lipoprotein receptor
Vir like m6A methyltransferase associated;Vir like m6A methyltransferase associated;Vir like m6A methyltransferase associated;Protein virilizer homolog isoform X1
Vitamin K-dependent protein C
Voltage-dependent anion-selective channel protein 3
von Willebrand factor A domain containing 8
VPS39 subunit of HOPS complex
VWFA domain-containing protein
WD repeat and FYVE domain containing 2

WD repeat domain 37
WD repeat domain 47
WD repeat domain 5
WH2 domain-containing protein
Wiskott-Aldrich syndrome protein family member
X-ray repair cross complementing 5;X-ray repair cross complementing 5;X-ray repair cross complementing 5;X-ray repair cross complementing 5;X-ray repair cross-complementing protein 5
Y-box binding protein 3
Yes1 associated transcriptional regulator
YTH N6-methyladenosine RNA binding protein 1;YTH N6-methyladenosine RNA binding protein 1;YTH N6-methyladenosine RNA binding protein 3;YTH N6-methyladenosine RNA binding protein 2;YTH N6-methyladenosine RNA binding protein 3;YTH N6-methyladenosine RNA binding protein 3;YTH N6-methyladenosine RNA binding protein 1;YTH N6-methyladenosine RNA binding protein 2;YTH N6-methyladenosine RNA binding protein 2;YTH N6-methyladenosine RNA binding protein 2
Zinc finger and BTB domain containing 20
Zinc finger CCCH-type containing, antiviral 1;Zinc finger CCCH-type antiviral protein 1 isoform 1;Zinc finger CCCH-type containing, antiviral 1;Zinc finger CCCH-type containing, antiviral 1
Zinc finger NFX1-type containing 1
Zinc finger protein 207;Zinc finger protein 207;BUB3-interacting and GLEBS motif-containing protein ZNF207 isoform b;Zinc finger protein 207;Zinc finger protein 207
Zinc finger protein 22
Zinc finger protein-like 1
Zw10 kinetochore protein;Zw10 kinetochore protein;Zw10 kinetochore protein;Zw10 kinetochore protein;Centromere/kinetochore protein zw10 homolog
Zyxin
